# Interferon signatures fuel B cell hyperactivity and plasmablast expansion in systemic lupus erythematosus

**DOI:** 10.1016/j.jaut.2025.103438

**Published:** 2025-05-26

**Authors:** Hugo J. van Dooren, Yemil Atisha-Fregoso, Annemarie L. Dorjée, Tom W.J. Huizinga, Meggan Mackay, Cynthia Aranow, René E.M. Toes, Betty Diamond, Jolien Suurmond

**Affiliations:** a Department of Rheumatology, Leiden University Medical Center, Leiden, the Netherlands; b Center for Autoimmune, Musculoskeletal and Hematopoietic Diseases, The Feinstein Institutes for Medical Research, Manhasset, NY, United States

**Keywords:** B cells, Plasma cells, Tolerance, Autoimmunity, Systemic lupus erythematosus

## Abstract

Systemic Lupus Erythematosus (SLE) is an autoimmune disease characterized by an array of autoantibodies, in particular anti-nuclear antibodies (ANA). The disease is also hallmarked by an expansion of plasmablasts (PB) and hypergammaglobulinemia. The mechanisms underlying this hyperactivity and its relation to autoantibody production is not clear. We aimed to characterize B cell hyperactivity in SLE to identify its underlying mechanisms.

Using deep phenotyping with spectral flow cytometry and scRNAseq, we demonstrate that a high frequency of PB relative to memory B cells marks a subgroup of SLE patients, particularly those with higher disease activity and positive for Sm/RNP autoantibodies. We identified the origin of this phenotype in a prominent IFN signature in PB and increased activation in the switched CD27^+^ memory B cell compartment. PB from this group of SLE patients displayed high levels of CD45RB and somatic hypermutation frequencies similar to memory B cells. Repertoire analysis revealed a highly polyclonal expansion of PB and skewing towards IgG1. B cell hyperactivity correlated with hypergammaglobulinemia, especially increased IgG serum levels.

In summary, we show for the first time a direct relationship between IFN and PB expansion in a subgroup of SLE patients. Increased activation and differentiation of class-switched B cells driven by IFN may directly underlie PB expansion and hypergammaglobulinemia. These results provide insight into the pathways leading to B cell hyperactivity and autoantibody production which may guide the tailoring of B cell- and IFN-targeted therapies.

## 1. Introduction

Systemic Lupus Erythematosus (SLE) is a systemic autoimmune disease characterized by autoantibodies and chronic inflammation. It is a heterogeneous disease that can affect multiple organs and clinical activity can fluctuate considerably [1]. The presence of antinuclear antibodies (ANA) is a hallmark of SLE. ANA consist of antibodies that can target a large variety of nuclear antigens [2]. The most well-known antigens are double-stranded DNA (dsDNA), and RNA binding proteins (RBPs) that can be further divided into Sm/RNP complex and SS-A/SS-B antigens. The presence of anti-dsDNA and anti-Sm are the most specific for SLE and are part of classification criteria [3]. Other ANA reactivities can be present in other autoimmune diseases [2].

ANA are produced by plasmablasts/plasma cells (PB/PC), which can arise through various B cell differentiation pathways [4]. The anti-chromatin and anti-RBP antibodies are often considered to arise through distinct pathways, anti-dsDNA titers fluctuate and are associated most strongly with disease flares and lupus nephritis, whereas most anti-RBPs are more stable [5,6]. Anti-dsDNA levels strikingly decreased following CD19 CAR T cell treatment while long-lived vaccination responses remained intact [7], suggesting an important role for extra-follicular B cell activation, at least for some of the ANAs. However, the exact pathways that are involved in the production of ANAs are not known. In particular, it is not clear whether and how global B cell alterations associate with antigenic specificity.

The most well-known feature of the B cell population in SLE is the presence of a high number of circulating PB, in particular of the IgG isotype [8–15]. Higher numbers of circulating PB are usually found in a subgroup of patients, and a high PB frequency or a high transcriptomic PB signature is associated with active disease or flares [11,12,14]. The mechanism for expansion of circulating PB is unclear. Such understanding is hampered by the sensitivity of PB to cryopreservation, making the analyses of PB more challenging in a rare disease setting. Inducing B cell hyperactivity through single gene variants or knockouts in mouse models often leads to PC expansion and hypergammaglobulinemia, while also inducing specific autoantibodies [16, 17]. Genetic variants leading to B cell hyperactivity have been reported for monogenic SLE in humans as well [18,19]. These gene variants often affect B cell signaling through the BCR or TLR, and as such, are expected to increase the response not only to self-antigens, explaining the heightened serum IgG levels. In line with this, altered function of B cell signaling as seen in SLE can augment B cell responses to both self- and foreign antigens [16–18]. B cell hyperactivity is causally involved in the loss of B cell tolerance and SLE. However, how PB expansion relates to specific ANA in human SLE is not clear.

Previously, we identified that patients with a high frequency of ANA + PB usually have a low frequency of ANA + CD27^+^ memory B cells [20]. This phenotype was particularly present in a subgroup of SLE patients, but we did not yet consider the global B cell alterations and the relationship to global PB expansion in these patients. Besides PB expansion, virtually all other B cell subsets can be altered in frequency in SLE, including double-negative 2 (DN2) cells, age- or autoimmunity-associated B cells (ABCs), activated naïve B cells, MZ-like B cells, and transitional B cells [21–24]. The mechanisms underlying alterations in B cell subsets and the relationship of each to PB expansion or differentiation is unclear. In addition, it is unknown which of the alterations in the distribution of the B cell compartment lead to increased antibody production, both for total serum immunoglobulins as well as for specific autoantibodies.

In this study we aimed to characterize B cell alterations in SLE and their relationship to immunoglobulin and autoantibody production, with the ultimate goal to identify features and mechanisms underlying B cell hyperactivity and autoantibody production. Our results reveal a novel distribution of the B cell compartment that associates with B cell hyperactivity, a phenotype that is particularly prominent in patients with active disease. This phenotype is characterized by a high PB to memory B cell ratio, increased activation in the switched memory compartment, and an IFN-associated polyclonal expansion of the PB compartment. A high PB to memory B cell ratio marks patients with increased ratios of IgG:IgM and Sm/RNP autoantibodies. Thus increased differentiation of switched B cells towards PB likely contributes to increased antibody production in SLE.

## 2. Methods

### 2.1. SLE patients and healthy subjects

#### Cohort 1:

Heparinized blood from 72 SLE patients (of which 36 were previously reported on) [20] and 14 healthy subjects was collected from The Feinstein Institute for Medical Research Rheumatology Specimen and Clinical Data Bank (Manhasset, NY, USA), and PBMCs were obtained using standard Ficoll procedure. PBMCs were used for flow cytometry (n = 72) and additional cell sorting for scRNAseq (n = 5) from the same patients. Serum was obtained from SLE patients at the time of PBMC isolation and was available for 69 patients. From 24 patients, a second sample of heparinized blood, was obtained to determine the stability of B cell phenotypes. Timepoints were more than 1 month apart (median 10; range: 1.5–26). SLE diagnosis was made clinical and fulfilled the 1997 revised ACR classification criteria [3]. SLE patients were excluded from the study if they received belimumab, rituximab, or cyclophosphamide 12 months prior to the study. Clinical data, including SLEDAI-2k and c-SLEDAI scores [25], were collected from the same timepoint as B cell analysis. Clinically active patients were defined as patients with a c-SLEDAI above zero.

#### Cohort 2:

Consisted of 77 SLE patients from the Rheumatology outpatient clinic at the Leiden University Medical Center (Leiden, The Netherlands) from which serum was available. Besides serum analysis being performed on all 77 SLE patients, cohort 2 was split into cohort 2A for flow cytometry analyses of fresh PBMCs (n = 37), cohort 2B for analysis of frozen PBMCs by scRNAseq (n = 4), cohort 2C for spectral flow cytometry analyses of fresh PBMCs (n = 17) and cohort 2D for spectral flow cytometry analyses of frozen PBMCs (n = 15). Five patients from cohort 2C and 2D were overlapping and measured in both fresh and frozen PBMCs obtained at the same time. Clinical disease activity data was collected for 17 SLE patients from cohort 2A. B cell phenotype was also measured in fresh PBMCs from 21 healthy subjects, and in frozen PBMCs from 8 Healthy subjects. One SLE patient was excluded for IgA analysis as it was deemed IgA-deficient. Information on which cohort was used in each main figure and replication figure is provided in Table S11.

Serum from 40 healthy donors were used to calculate the normal values and cut-offs for ELISA, and were obtained from the LUMC Voluntary Donor Service.

Patient characteristics from both cohorts are described in Tables S1 and S2. The study with SLE patients and healthy subjects from the USA was approved by the Northwell Health Institutional Review Board, and the study with PBMCs from SLE patients and healthy subjects from Leiden were approved by the Leiden-Den Haag-Delft Medical Ethical Committee. The use of serum samples from healthy subjects was approved by the LUMC-biobank organization. All subjects gave written informed consent.

### 2.2. Flow cytometry

PBMCs were stained for surface markers using fluorescent antibodies diluted in HBSS +2 % FCS (Cohort 1) or PBS containing 1 % BSA (Cohort 2). eFluor 506–labeled fixable viability dye (eBioscience) was added during staining with cell surface antibodies. For spectral flow cytometry, antibody cocktails were prepared using Brilliant Stain Buffer (BSB) Plus (BD) and monocyte blocker (Biolegend) was added to prevent aspecific binding of tandem dyes. For samples with surface staining alone, cells were washed and left unfixed or were fixed with 1 % PFA. For samples with intracellular staining, cells were washed and fixed and per-meabilized with Foxp3 transcription factor fixation/permeabilization kit (eBioscience) according to manufacturer’s instructions. Antibodies used in this study are shown in Supplementary Tables S8 and S9.

Conventional flow cytometric acquisition was performed on a Fortessa (BD). FACS sorting was performed on FACS Aria or Aria Sorp sorters (BD). Analysis of conventional flow cytometry and FACS sorting was performed using FACS Diva (BD) and FlowJo software. Spectral flow cytometric acquisition was performed on a 5-laser Aurora (Cytek).

### 2.3. scRNAseq library preparation and sequencing

PB (CD3/14/FVD-CD19+CD27++CD38++) were isolated by FACS sorting, washed in HBSS or PBS + 0.04 % non-acetylated molecular grade BSA (Thermo), centrifuged at 1000×*g* for 5 min, and resuspended in the same buffer. Cell suspensions were processed through the Chromium Single-Cell 5′ RNA-seq system (10x Genomics), according to the manufacturer’s protocols. In the first experiment, cells from 4 patients in Cohort 1 were run on separate lanes using Chromium Single Cell 5′ Library & Gel Bead Kit v1.1. In a second experiment, cell hashtags (Biolegend Totalseq antibodies) were used to multiplex frozen cells from 5 patients from Cohort 2B on a single lane using Chromium Single Cell 5′ Library & Gel Bead Kit v1.1 as well as the Single Cell 5′ Feature Barcode Library Kit (10x Genomics). After cDNA amplification, BCR target enrichment was performed using the Chromium Single Cell V(D)J Enrichment Kit for Human B cells (10x Genomics). Sequencing of libraries from the first experiment was performed on a HiSeq system (Illumina) with 2 × 150bp reads. Sequencing of the second experiment was performed on a partial lane of the illumina NovaSeq6000 at a 151-16-9-151 bp run configuration. The target sequence depth was 25K reads/cell for Gene expression, 5K reads/cell for BCR, and 2.5K reads/ cell for Hashtag oligo. Sequencing saturation was >70 % in each sample.

### 2.4. ELISA

384-well Clear Flat Bottom Polystyrene High Bind Microplates (Corning) were coated overnight at 4C with 1 μg/ml UltraPure™ Salmon Sperm DNA Solution (Thermo), 0,5 μg/ml non-recombinant bovine nucleosomes, 0,5 μg/ml non-recombinant bovine histones, 0,8 μg/ml non-recombinant bovine Sm, 0,6 μg/ml recombinant human U1snRNP-68/70 kDa, 0,8 μg/ml non-recombinant bovine Sm/RNP70 complex, 0,5 μg/ml 60-kDa recombinant human SS-A or 1,1 μg/ml recombinant human SS-B (all from Diarect) diluted in PBS.

All washing steps were performed by washing 4 times with 0.05 % Tween20 (Sigma) in PBS using the Berthold Zoom HT LB 920 Plate Washer. Plates were washed and subsequently blocked with 1 % BSA (Sigma) and 50 mM Tris (Roche, pH = 8,0) in PBS for 1 h at RT. Subsequently, the plates were washed and the wells were incubated with serum, diluted 1:200 in PBS/0.05 % Tween20/1 % BSA/Tris (pH = 8,0), for 1 h at RT. After the incubation, the ELISA plates were washed and incubated with 1 μg/ml anti-IgG horse-radish peroxidase (Bethyl) and, subsequently, washed and incubated with ABTS (Sigma-Aldrich) with 1:2000 hydrogen peroxide (Merck), All plates were measured at 415 nm on the SpectraMax i3x Multi-Mode Microplate Reader. ANA standards consisted of pooled sera from positive SLE patients for each reactivity. In-house ANA ELISAs were validated using SLE serum samples with known reactivities obtained from the anti-ENA SLE profile 2 ELISA kit (Euroimmune) or clinical diagnostic assays (Fig. S13). All ELISAs reached a positive and negative predictive value of >0.8.

Total IgG, IgM and IgA ELISAs were performed by coating 384-well Clear Flat Bottom Polystyrene High Bind Microplates with 10 μg/ml goat anti human IgG/IgM and IgA Fc (Bethyl) diluted in mQ water with 0,1 M Na_2_CO_3_/NaHCO_3_ (Merck, pH = 9,6) for 1 h at RT. Same protocols were used as for ANA ELISAs except serum samples were diluted 800.000x for total IgG, 40.000x for total IgA, and 20.000x for total IgM. Commercial standards with quantified IgM and IgA levels (Bethyl) or IgG (Southern Biotech) were used for quantification.

ANA and total Ig levels were expressed as aU/ml or mg/mL, respectively, based on the intrapolation from the standards using a 5-parameter logistic curves. For autoantibodies, samples were considered positive when they were above the cut-off, determined by the mean value + 4x std of 40 healthy donors. One and two healthy donors were excluded for determination of the cut-off for anti-SS-A and anti-Sm, respectively, because they were above the cut-off of the rest of the healthy donors, and had a large impact on the sensitivity of the assay determined by commercial ELISA.

### 2.5. Statistics

Statistical analysis was performed in GraphPad version 10.2.3. P-values were considered statistically significant if they were below 0.05. Fisher’s exact test was used to analyse categorical data. For continuous data, Mann Whitney U tests were used to compare two groups, except qPCR which was analysed using Student’s t-test after log-normalization. Kruskal-Wallis was used to compare multiple groups, with false discovery rate (FDR) as posthoc test calculated using the Two-stage linear step-up procedure of Benjamini, Krieger and Yekutieli. Two-way ANOVA was used for statistical analysis of grouped data, with FDR as posthoc test calculated using the Two-stage linear step-up procedure of Benjamini, Krieger and Yekutieli. Correlations were analysed using simple linear regression. For multiple linear and logistic regression, continuous variables were log-transformed and converted to Z-scores for standardization.

Cut-offs of %PB and PB/M ratio were based on data from 14 healthy donors from Cohort 1, 21 donors from Cohort 2 (fresh) and 8 donors from Cohort 2 (frozen), calculated using Q3 + 1.5*IQR. One healthy donor from the Feinstein Institute and 2 from the Leiden cohort were excluded for calculation of the cutoffs as it exceeded the cut-off and were thus deemed outliers. These subjects were only excluded for determination of the cut-offs but remained present for statistical comparison of healthy subjects versus SLE patients. The resulting cutoffs were 2.75 for %PB, 0.105 for PB/M ratio on fresh cells, and a PB/M ratio of 0.035 for frozen cells. The latter was 3-fold lower on frozen cells compared to fresh cells due to specific loss of PB after freezing (Fig. S4A). Using 5 SLE samples with paired fresh and frozen data, the PB/M ratio was also decreased approximately 3-fold (Fig. S4B). For absolute PB count, healthy donor count data was unavailable, therefore these groups were divided based on the median of PB counts in SLE patients in cohort 1 (1.91 cells/uL).

Additional methods (5′RACE PCR, RNA isolation and qPCR) and data analysis methods for spectral flow cytometry, scRNAseq, and repertoire analysis are provided in Supplementary Methods.

## 3. Results

### 3.1. A high PB to memory B cell ratio characterizes a subgroup in SLE with high disease activity

**Fig. 1.**
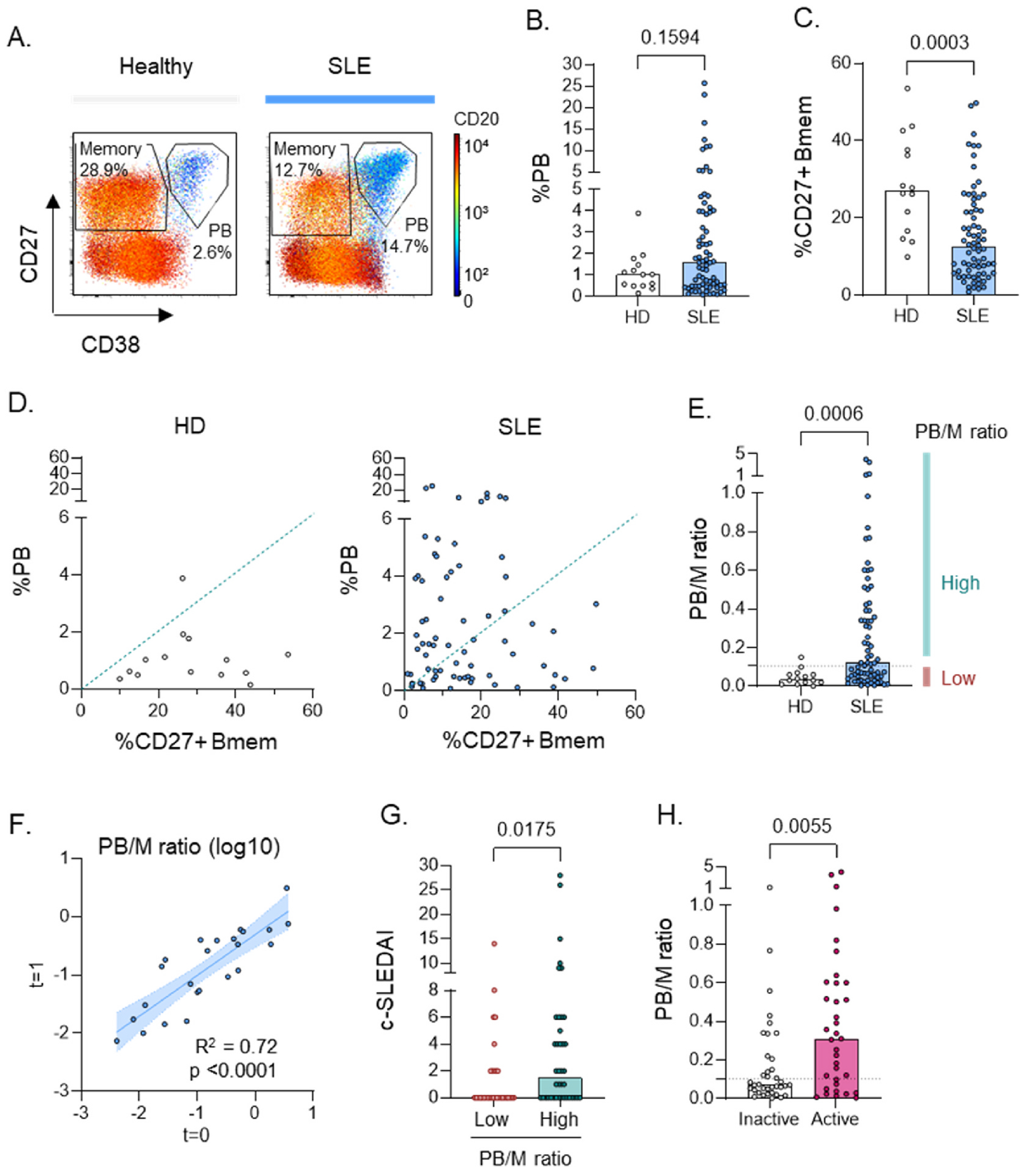
A high PB to memory B cell ratio characterizes a subgroup in SLE with high disease activity. B cell phenotypes of SLE patients (n = 72) and healthy donors (n = 14) from cohort 1 were analysed using flow cytometry. A) Representative examples of PB and Bmem gating. B-C) Percent PB and CD27^+^ Bmem among total B cells. D) Scatterplot of %PB and %CD27+ Bmem with the dashed line representing a PB/M ratio of 0.105. E) Ratio of PB over CD27^+^ Bmem (PB/M). F) Correlation of PB/M ratio over time in SLE patients whose B cell phenotype was measured twice (n = 24). G) c-SLEDAI in SLE patients with a low versus high PB/M ratio. H) PB/M ratio in patients with clinically inactive versus active (c-SLEDAI>0) disease. Each dot indicates an individual, and the bars represent the median. P values were calculated using Mann-Whitney test (B,C,E,G,H), or linear regression (F).

Several studies report increased numbers of circulating PB in SLE. In these studies, the relative frequency of PB out of total B cells (%PB) as well as the absolute PB count are frequently found to be increased [11, 12,14]. Using principal component analysis we previously found a distinct SLE patient group with a high frequency of ANA + PB relative to the frequency of ANA + CD27^+^ memory B cells (Bmem) [20]. We now investigated whether a similar PB to memory ratio was also observed in the total B cell compartment. Consistent with literature, we observed a trend for a higher frequency of PB (CD27^hi^CD38^hi^CD20^lo^) within CD19^+^ B cells, particularly visible in a subset of SLE patients (Fig. 1A and B). As we and others observed before, SLE was also characterized by a significantly reduced frequency of CD27^+^ Bmem (CD27+CD38^lo^CD20+) (Fig. 1C) [16,20]. Patients with a high frequency of PB tended to have a lower frequency of CD27^+^ Bmem (Fig. 1D). The ratio of PB to CD27^+^ Bmem (PB/M ratio) in SLE patients was significantly increased compared to healthy donors (Fig. 1E). As a subset of patients exhibited this phenotype, we divided SLE patients into subgroups with a low and high PB/M ratio, with a cutoff based on the healthy donors (Q3 + 1.5*IQR). The PB/M ratio was highly stable over time as repeated measurements approximately 1 year apart available in the US cohort (cohort 1; n = 24) revealed a strong correlation (Fig. 1F). These findings were replicated in an independent cohort in Europe (cohort 2A; Fig. S1).

Analysis of patient characteristics of the “Low PB/M″ and “High PB/ M″ groups in cohort 1 (Table S1 and S2) revealed trends for a higher frequency of male/female (OR: 6.576; 95 %CI: 1.034–76.14) and self-reported African-American race (OR: 2.778; 95 % CI: 1.104–6.827) in the high PB/M group, though these were not statistically significant and not reproduced in the replication cohort 2A. The only significant difference was observed for Asian race, which was more abundant in the low PB/M group, though this was based on small numbers of patients in cohort 1. We next addressed whether patients with a high PB/M ratio display a different clinical profile. Patients with a high PB/M ratio had a higher SLEDAI-2k disease activity score and more frequently moderate to high disease activity (Fig. 1G and H, Table S3). Clinical activity was observed across different disease manifestations, though not statistically significant for most symptoms (Table S4). The exception was hematological disease activity, in which patients with a high PB/M ratio more frequently had leukopenia and lymphopenia, but not thrombocytopenia (Fig. S2A–D). Patients with a high PB/M ratio also displayed decreased levels of complement C4, but not C3 (Fig. S2E and F). No difference in disease activity was observed when analyzing SLE patients by frequency or absolute numbers of PB (Fig. S2G and H). No clear differences with regards to medication were observed (Table S1 and S2).

Though the PB/M ratio was relatively stable over time (Fig. 1E), we also analysed whether a change in PB phenotype associated with a change in disease activity. No clear correlation between a change in disease activity (delta SLEDAI-2k) and change in PB parameters (delta PB/M ratio or delta %PB) was observed (Fig. S2I–L), possibly due to low patient numbers (n = 24).

Overall, these results suggest a stable phenotype of high PB/M ratio that is associated with a higher risk for clinically active disease.

A high frequency of PB relative to memory B cells (PB/M) is present in a subgroup of SLE patients with a higher disease activity. This more strikingly differentiates between SLE patients and healthy subjects than the frequency or number of PB.

### 3.2. Increased activation within switched B cell compartments

**Fig. 2.**
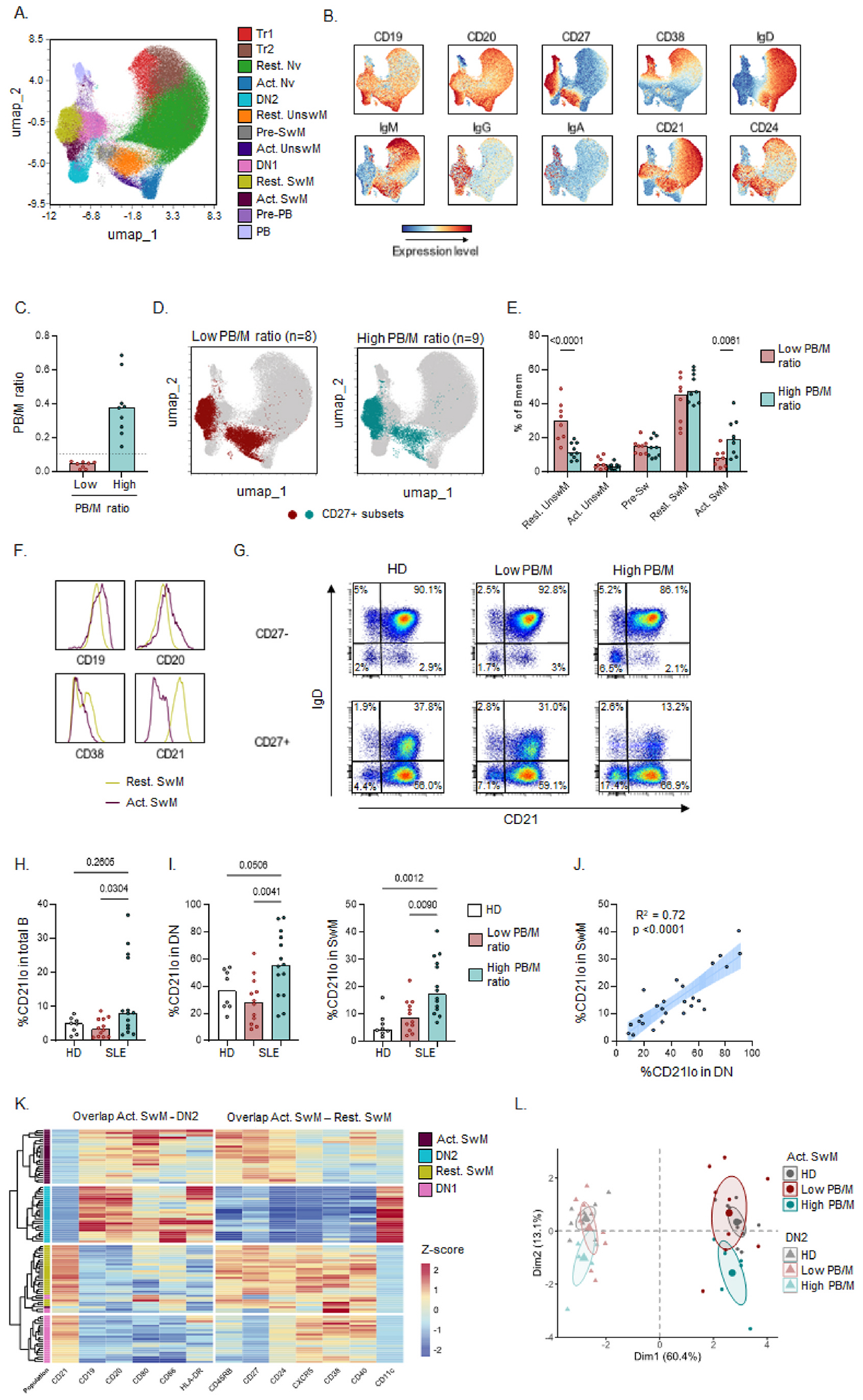
Increased activation within switched B cell compartments. High dimensional spectral flow cytometry was used for detailed B cell phenotyping within fresh PBMCs (A–F) from SLE patients (cohort 2C; n = 17) and a second cohort of frozen PBMCs (K–L) from SLE patients (cohort 2D; n = 15) and healthy donors (HD; n = 10). Data from both experiments was pooled in H-L, after calculating the average for 5 samples present in both. Additional data on both cohorts is provided in Figs. S3 and S4. A) Live B cells were clustered using FlowSOM and visualized in UMAP. B) Normalized expression levels of B cell markers in UMAP plot. C) PB/M ratio in this dataset. D) Projection of CD27^+^ Bmem cells on the UMAP plot. E) %clusters within the CD27^+^ Bmem cells in SLE patients with a low versus high PB/M ratio. F) Histograms of key markers defining the Act SwM cluster. G) IgD and CD21 expression in CD27 ^−^ and CD27^+^ B cells. H) %CD21^lo^ within total B cells. I) %CD21lo within switched B cells (DN and SwM subsets). J) Correlation of %CD21lo within DN and SwM B cells in SLE patients. K) Clustered heatmap of expression level of activation markers in 4 switched B cell subsets in the second dataset. Expression level is represented as a z-score of the scaled median fluorescence intensity per subset per sample. L) Principal component analysis of all B cell markers from panel K. The percentage indicated on the axis is the % of variance explained by that principal component. The ellipse depicts the 95 % confidence interval. Each dot indicates an individual, and the bars represent the median. P values were calculated using Mann-Whitney test (C), Kruskal-Wallis with FDR posthoc test (H, I), simple linear regression (J) or Two-way ANOVA with FDR posthoc test (E).

To obtain further understanding of the alterations of B cell populations in SLE, we performed detailed spectral flow cytometry. Based on unsupervised clustering with 8 B cell markers (CD19, CD20, CD27, CD38, CD21, CD24, IgD, IgM), we identified 13 B cell subsets (Fig. 2A and B, Fig. S3A). Clusters were assigned to B cell populations according to published definitions [26]. Using the same cutoff (0.105) for the PB/M ratio to subset SLE patients (Fig. 2C), we first analysed the distribution of subsets within the CD27^+^ memory compartment (Fig. 2D and E), revealing a strong decrease in resting unswitched memory B cells (CD27+CD38^lo^CD21+CD24+IgD + IgM+) in SLE patients with a high PB/M ratio. These cells have also been referred to as marginal zone-like cells [16,26,27]. Despite the overall reduction in CD27^+^ memory B cells, an increase in the activated switched memory compartment was observed. These cells were characterized by increased expression of CD19, CD20, intermediate CD38, and low CD21 expression compared to resting switched memory B cells (Fig. 2F). Within the CD27^−^ B cell subsets a significant decrease in resting naïve B cells was observed in patients with a high PB/M ratio (Fig. S3B). Besides a relative increase in activation within the SwM compartment, Pre-PB, cells with an intermediate phenotype between Act SwM and PB, were also increased in patients with a high PB/M ratio (Fig. S3C and D).

To further confirm the findings described above, we next generated an additional dataset using a more detailed spectral flow cytometry panel including several activation markers on frozen PBMCs [16]. Despite the relative reduction of PB in frozen cells (Fig. S4A and B), the main differences in B cell subset distribution in SLE compared to HD were confirmed (Fig. S4C–H). Though no significant differences were observed in the percentages of DN2 out of total B cells in both datasets, when combining both datasets and focusing on the CD21^lo^ cells within each subset, both CD27^−^ and CD27^+^ cells displayed increased percentages of CD21^lo^ cells, most prominently in the switched compartments (DN and SwM respectively) (Fig. 2G–I). These subsets represent DN2 and activated SwM B cells, respectively. The frequency of CD21^lo^ cells within these subsets was highly correlated (Fig. 2J), suggesting that activation across switched B cells is common in SLE patients, in particular those with a high PB/M ratio.

Since CD21^lo^ cells have been implicated in SLE before, we analysed the expression level of activation markers in the different switched B cell clusters with low CD21 expression and their CD21^+^ counterparts (DN2 vs DN1, and Activated SwM vs Resting SwM). Hierarchical clustering revealed that Act SwM cells have similarities with DN2 cells (decreased CD21, increased CD19, CD20, CD80, CD86, HLA-DR) confirming their activated status, even though sharing marker expression with conventional CD27^+^ memory B cells (increased CD45RB, CD27, CD24, CXCR5, CD38, CD40 and low CD11c) (Fig. 2K). High CD45RB expression has been previously linked to a germinal center origin [28], and CD45RB expression in PB was high and comparable to that of CD27^+^ memory B cell subsets in both patient groups (Fig. S5A). Using principal components analysis of activation markers, the phenotype of Act SwM in patients with a high PB/M ratio was distinct from that of patients with a low PB/M ratio (Fig. 2L), and was characterized by decreased expression of CD21 and increased expression of CD38, CD86, CD11c, and a trend towards increased expression of CD40 and CD80 (Fig. S5B and C). Together, these data indicate that both the frequency and degree of activation of CD21^lo^ switched memory B cells are increased in patients with a high PB/M ratio. Though DN2 cells displayed a less divergent phenotype, CD86 and CD11c were also expressed at significantly higher levels in DN2 cells from patients with a high PB/M ratio (Fig. S5B and C). Using trajectory analysis starting at Tr1 cells, the DN2, Act SwM and Pre-PB populations were those closest to PB (Fig. S3E–G). Thus, a high PB/M ratio is related to B cell hyperactivity in switched B cells with skewing of B cells towards the most activated and differentiated subsets.

### 3.3. Increased proliferation and IFN signature underly PB expansion in patients with a high PB/M ratio

**Fig. 3.**
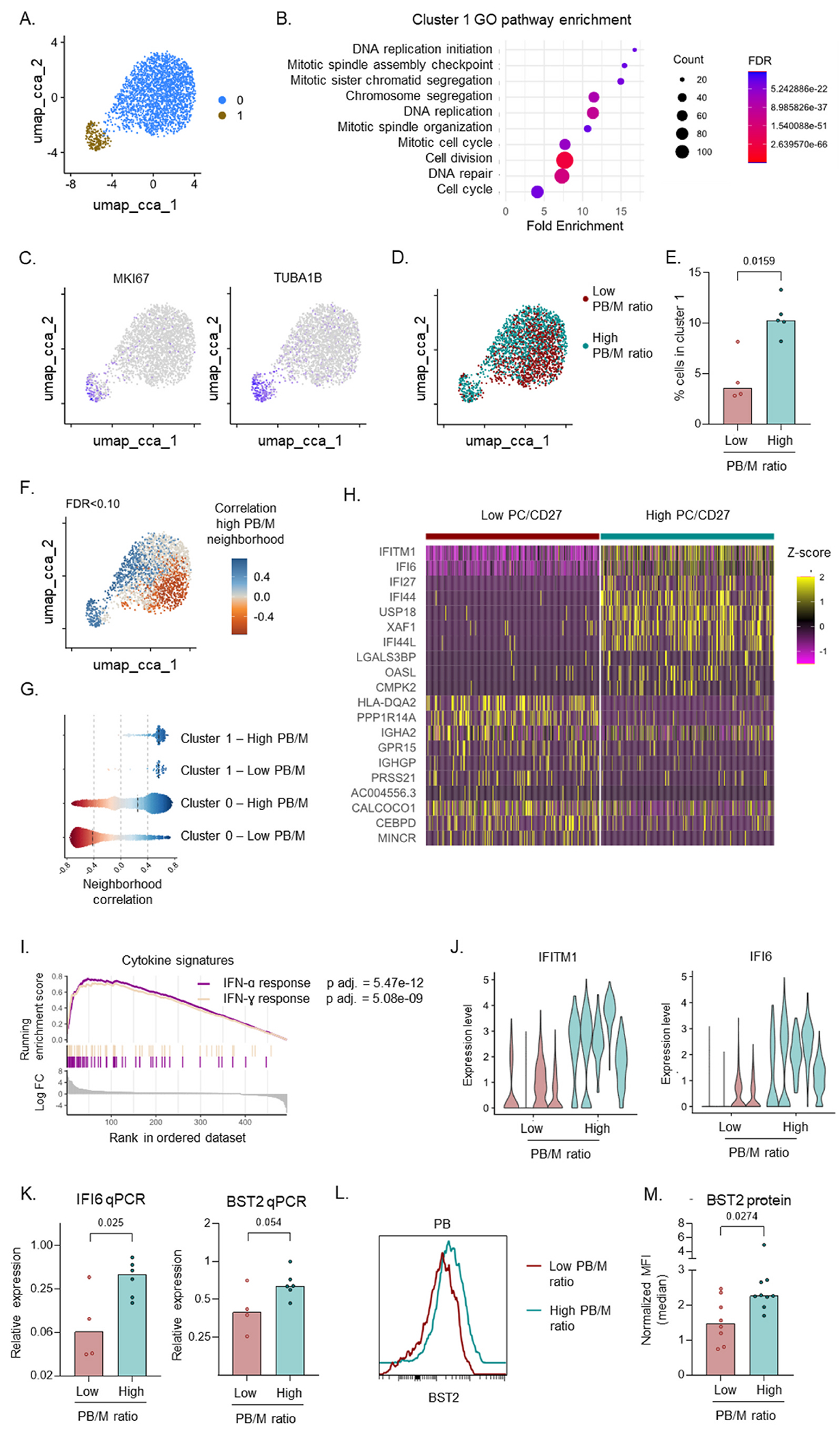
Increased proliferation and IFN signature underly PB expansion in patients with a high PB/M ratio. scRNA-seq was performed on sorted PB from 9 SLE patients, in 2 independent experiments from cohort 1 and cohort 2B. A) UMAP using canonical correlation analysis for integration of both datasets with 2 clusters identified using unsupervised clustering. B) Pathway enrichment in genes upregulated in cluster 1. C) Expression level of MKI67 and TUBA1B on UMAP. D) Distribution of cells in patients with a low versus high PB/M ratio. E) % cells in cluster 1 between the two patient groups. F,G) Results of association test for a high PB/M ratio using CNA (CNA global P = 0.030): each cell with an FDR> 0.010 is colored according to its neighborhood coefficient, with blue indicating a positive correlation and red indicating a negative correlation (F), and each cell is split per patient group and per cluster (G). H) Heatmap of top 10 differentially expressed genes (adjusted p value < 5*10e-5, highest fold change) between patients with a low versus high PB/M ratio. 250 cells from each group are displayed. I) GSEA for cytokine signatures using differentially expressed genes (adjusted p value < 0.05; ordered by log fold change) between the two patient groups. J) Expression level of IFI6 and IFITM1 in patients with a low versus high PB/M ratio. Each patient is displayed as individual “violin”. K) RNA expression of IFI6 and BST2 in sorted PB, determined by qPCR. Relative expression was calculated based on two housekeeping genes. L,M) BST2 protein expression on PB was determined using spectral flow cytometry as described in Fig. 2. L) Representative example. M) Normalized expression of BST2 in patients with a low versus high PB/M ratio. Each dot in the barographs indicates an individual, and the bars represent the median. P values and FDR values were calculated using DAVID functional annotation (B), Mann-Whitney test (E,M), CNA (F), GSEA (I), or student’s T-test on log-normalized expression (K). (For interpretation of the references to color in this figure legend, the reader is referred to the Web version of this article.)

To identify the mechanism underlying PB differentiation in these patients, we performed scRNAseq of FACS sorted PBs (CD19+CD27++CD38++) from SLE patients with a low (n = 4) versus high (n = 5) PB/M ratio, in 2 independent experiments. After QC filtering based on number of genes detected per cell, percentage of mitochondrial genes, contaminating cells (non-PB) and doublets (see Methods), there were a total of 1290 and 1197 PB analysed in the two experiments respectively. Using unsupervised clustering and visualization, the clusters and differentially expressed genes largely overlapped between the datasets (data not shown), and were correlated to the patient B cell phenotype in both datasets. We therefore proceeded with an integrated analysis of both datasets. Using unsupervised clustering, 2 distinct clusters could be identified (Fig. 3A). Genes upregulated in cluster 1 were associated with proliferation/cell division (Fig. 3B,C, Fig. S6A and B), and this cluster was increased in frequency in patients with a high PB/M ratio (Fig. 3D and E). Importantly, besides this increased abundance in cluster 1, covarying neighborhood analysis revealed that cells from patients in each group were closely related to cells from their own group despite being represented in one cluster (Fig. 3F and G). Differential gene expression revealed increased expression of IFN-stimulated genes in PBs from patients with a high PB/ M ratio (Fig. 3H,I, Fig. S6C). Whereas IFN-stimulated genes between type I IFN and IFN-gamma largely overlap, enrichment in transcription factor binding sites for IRF7 and ISRE (Fig. S6D), as well as expression levels of IFN receptor units (Fig. S6E) suggest this signature is most likely related to type I IFN.

The increased IFN signature was observed in each individual patient from the high PB/M group (Fig. 3J) and was confirmed in additional patients by qPCR (Fig. 3K). Furthermore, increased protein expression of BST2, an IFN-regulated cell surface marker [29], was confirmed on PBs by spectral flow cytometry (Fig. 3L and M). Within the PB/M high patient group, high BST2 expression was shared among all CD21^lo^ B cell subsets (activated naïve, DN2, activated unswitched and activated switched memory) as well as pre-PB and PB (Fig. S7A). However, high expression of BST2 in CD21lo subsets was also found in healthy donors and SLE patients with a low PB/M ratio. No significant differences in BST2 expression were found between groups for CD21lo subsets (Fig. S7B). In contrast, Pre-PB and PB showed significantly increased BST2 expression in SLE patients with a high PB/M ratio (Fig. S7C). This suggests that IFN is highly associated with accelerated differentiation of PBs in patients with a high PB/M ratio.

### 3.4. IgG1 skewing and highly polyclonal BCR repertoire in PB

**Fig. 4.**
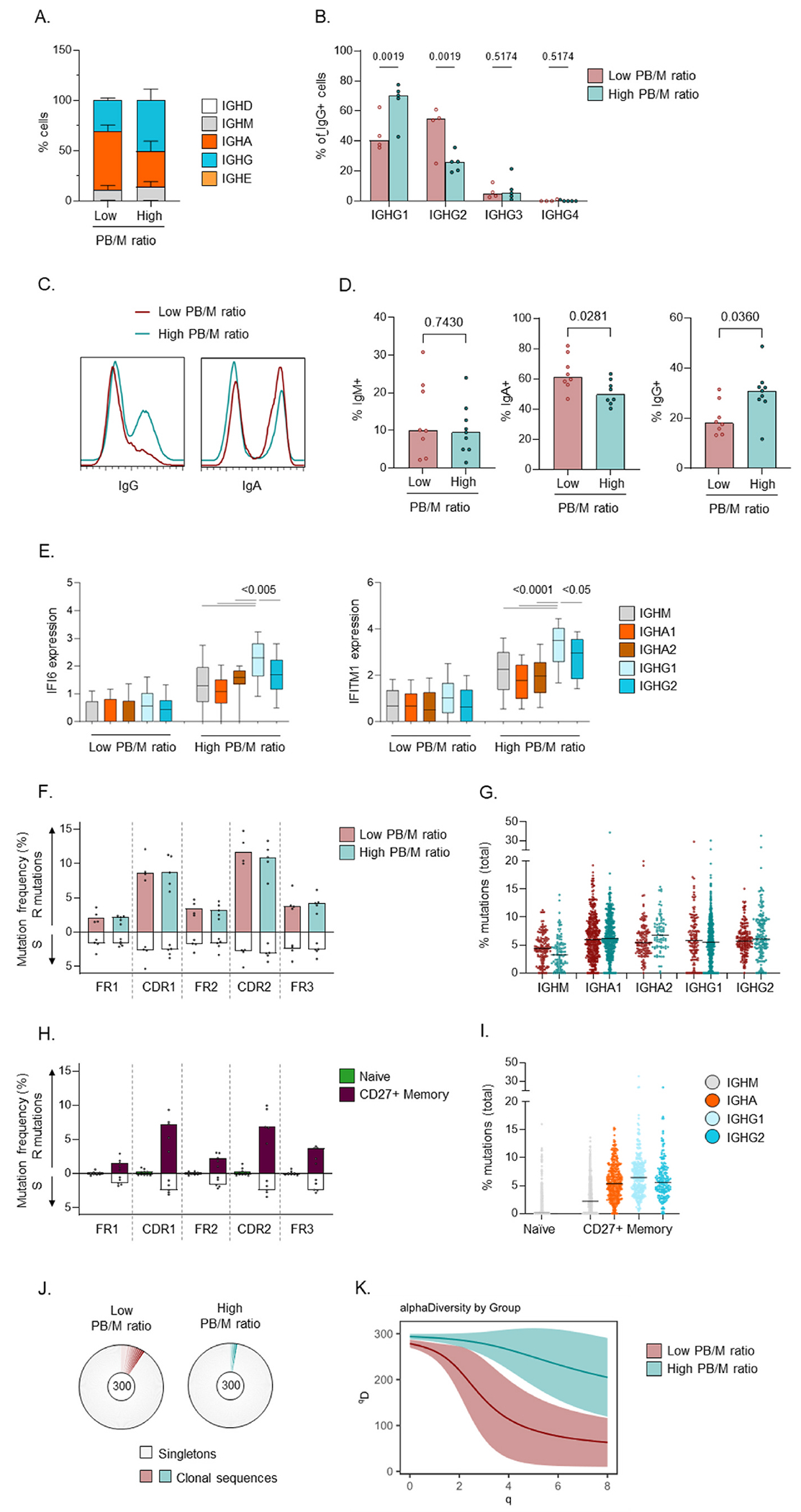
IgG1 skewing and highly polyclonal BCR repertoire in PB. BCR repertoire was determined using results from scRNA-seq (Fig. 3) from 9 SLE patients, in 2 independent experiments from cohort 1 and cohort 2B. A) Frequency of IGH isotypes in PB from patients with a low versus high PB/M ratio. B) IGHG subclass distribution within IgG + cells in both patient groups. C,D) Expression of IgM, IgA, and IgG in PB was determined using spectral flow cytometry as described in Fig. 2. C) Representative examples for IgA and IgG. D) Percent of each isotype in PB from patients with a low versus high PB/M ratio. E) scRNAseq expression level of IFI6 and IFITM1 in cells from each IGH isotype/subclass, separated by patient group. IGHD, IGHG3, and IGHG4 cells were not analysed due to low number of cells (panel A). F,H) % replacement € and silent (S) mutations, per region, in PB from patients with a low versus high PB/M ratio (F) and in naïve and CD27^+^ memory B cells from SLE patients (H). G,I) Total % mutations in PB by Ig subclass in PB (G) and in naïve and CD27^+^ memory B cells from SLE patients (I). J) Expanded clones and singletons in PB from both patient groups. scRNAseq PB data was downsampled to 100 cells/patient and the total of 3 patients is shown. K) Smoothed alpha diversity (qD) curve over a range of diversity orders (q) in data from panel J. Data in are shown as mean +SEM (A), as median across individual patients (B,D,F,H), as boxplot with interquartile ran€(E), as individual cells with the median across all cells (G,I), or as mean with the 95 % confidence interval K). P values were calculated using Two-way ANOVA with FDR posthoc test (B,F-G), Mann-Whitney test (D), or One-way ANOVA with FDR posthoc€st (E). No significant differences were found between patients in F,G.

We next analysed the BCR repertoire obtained through scRNAseq to obtain insight into the cellular origin of PB in patients with a high PB/M ratio. First, we discovered that patients with a high PB/M ratio displayed a skewing in the isotype distribution, with decreased IgA and increased IgG abundance (Fig. 4A). In addition, we observed increased IgG1 and decreased IgG2 within the IgG + cells in this group (Fig. 4B). Decreased IgA and increased IgG abundance in PB was confirmed in spectral flow cytometry (Fig. 4C and D). Interestingly, IgG1+ cells displayed the most prominent IFN signature with highest expression of IFN-stimulated genes IFI6 and IFITM1 (Fig. 4E). IgG1 cells had a similar expression level of IFNAR1/2 as other subclasses (Fig. S8A), suggesting the increased IFN signature is due to their local activation/differentiation by IFN.

No obvious skewing of V gene usage was observed (Fig. S8B). No difference in somatic hypermutation (SHM) was observed between the groups, whether analysed by location in the V-gene or by isotype/subclass (Fig. 4F and G). The frequencies of SHM were similar to those observed for memory B cells (Fig. 4H and I). Together with the high R/S ratio in CDR regions this suggests that PB in both groups originate from cells that underwent selection processes, most likely in the germinal center. Analysis of clonality revealed increased diversity in patients with a high PB/M ratio compared to those with a low PB/M ratio (Fig. 4J and K).

Together, these analyses indicate that PB in patients with a high PB/ M ratio arise through IFN-driven polyclonal differentiation and/or expansion of IgG1+ PB likely derived from post-germinal center cells.

### 3.5. B cell hyperactivity results in hypergammaglobulinemia

**Fig. 5.**
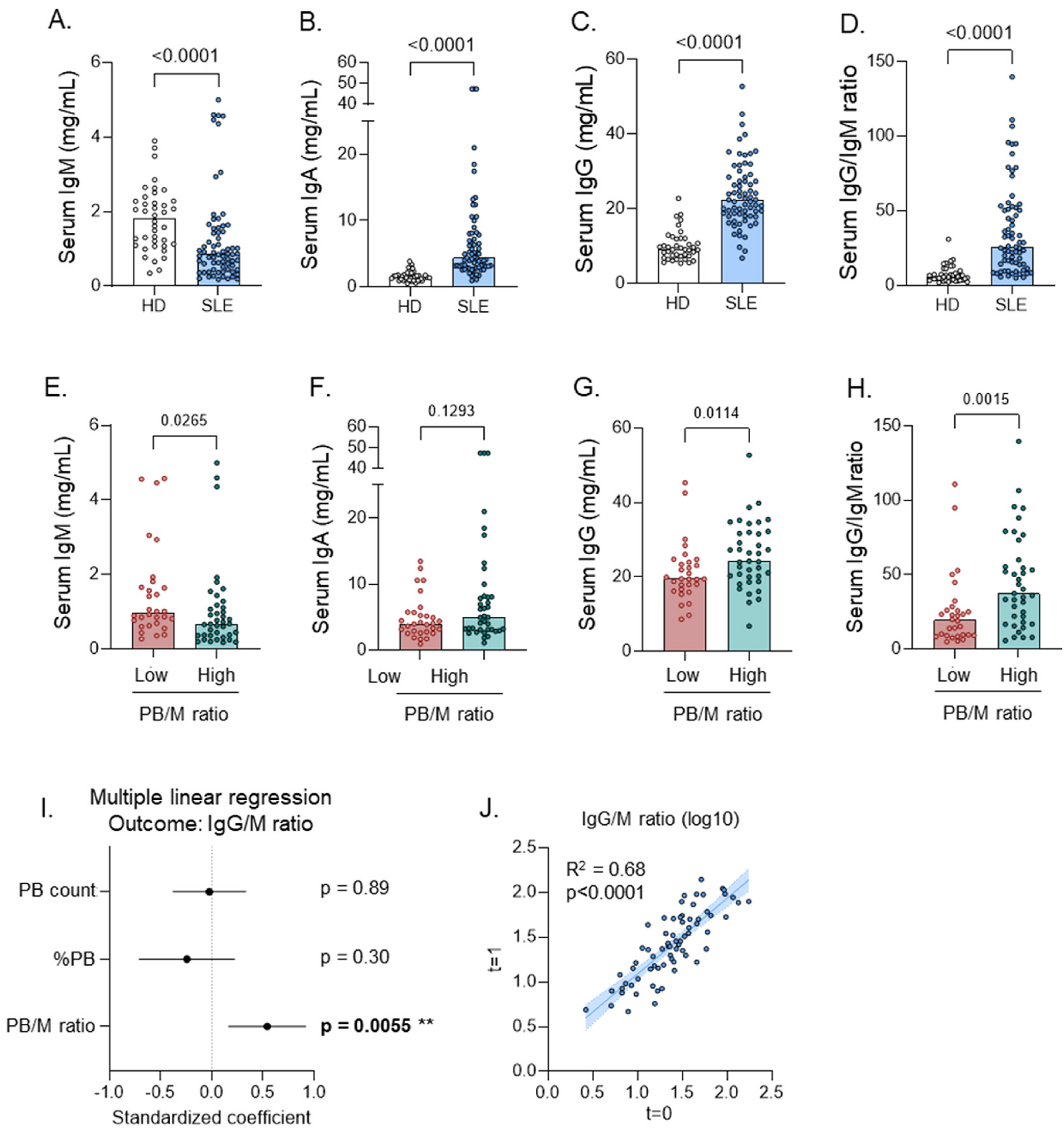
B cell hyperactivity results in hypergammaglobulinemia. Serum antibodies were measured in SLE patients (n = 69) from cohort 1 and healthy donors (n = 40) using ELISA. SLE patients were split into two groups based on PB phenotype shown in Fig. 1 A-H) Serum levels of total IgG, IgA, and IgM, and IgG:IgM ratio in SLE patients and healthy donors (A–D) and SLE patients split according to PB/CD27 group (E–H). I) Forest plot of multiple linear regression model with IgG:IgM ratio as outcome, and the Z-score of PB count, %PB, PB/M ratio, and cohort as predicting variables. J) Correlation of serum immunoglobulin levels in SLE patients measured at two timepoints ~1 year apart (n = 72). Each dot indicates an individual, and the bars represent the median (A-H,J). P values were obtained using Mann-Whitney test (A–H), multiple linear regression (I), or simple linear regression (J).

Hypergammaglobulinemia is often regarded as a sign of B cell hyperactivity. We observed decreased levels of IgM and increased levels of IgA and IgG in SLE compared to healthy controls, resulting in a high ratio of IgG:IgM in SLE patients (Fig. 5A–D). These features were most pronounced in SLE patients with a high PB/M ratio (Fig. 5E–H). These findings were replicated in cohort 2A (Fig. S9A–H).

Importantly, serum immunoglobulin changes were less pronounced or not significant when patients were grouped based on the frequency of PB or the PB count (Fig. S10A–H), or on the frequency of memory B cells (data not shown). Furthermore, in a multiple linear regression model with continuous values of the PB/M ratio, frequency of PB and PB count as predicting variables (thus independent from specific cut-offs used to group patients), only the PB/M ratio could significantly predict IgG:IgM ratios (Fig. 5I). Like the PB/M ratio, levels of serum immunoglobulins significantly correlated over time, in particular for the IgG/IgM ratio (Fig. 5J, Fig. S10I–K).

Together, these data suggest that B cell hyperactivity in the IgG B cell compartment, reflected in a high PB/M ratio, results in stable hypergammaglobulinemia in SLE.

### 3.6. B cell hyperactivity is specifically associated with the presence of Sm/ RNP autoantibodies

**Fig. 6.**
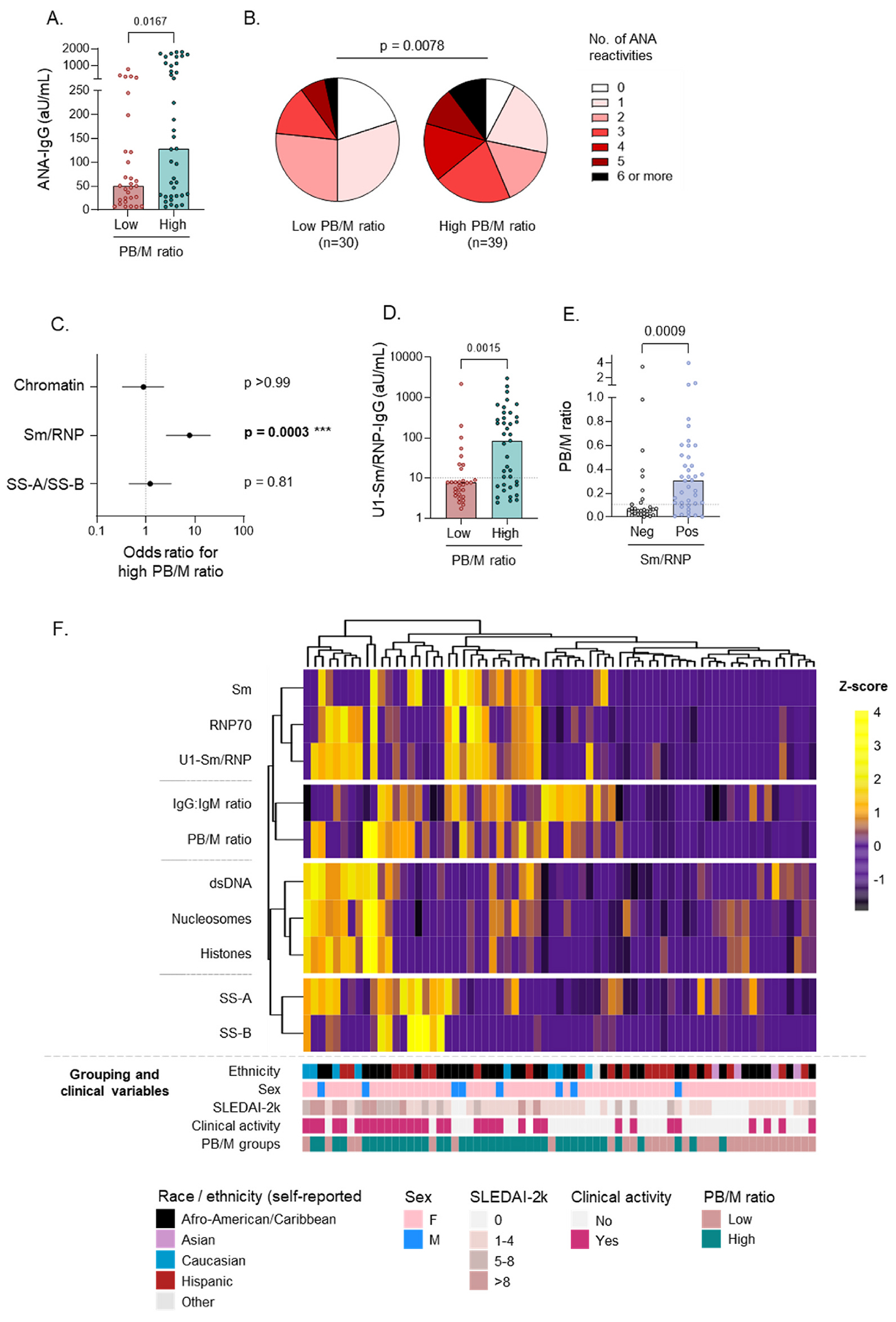
B cell hyperactivity is specifically associated with the presence of Sm/RNP autoantibodies. Serum autoantibodies were measured in SLE patients (n = 69) from cohort 1 using ELISA. Healthy donors (n = 40) were used as negative controls. Autoantibody reactivities were categorized into three main antigen groups: chromatin (dsDNA, nucleosomes, histones); Sm/RNP (Sm, RNP70, U1-RNP complex), SS-A/B (SS-A, SS-B). SLE patients were split into two groups based on PB phenotype shown in Fig. 1. A) Level of ANA-IgG in SLE patients split according to PB/M ratio. B) Number of IgG-ANA reactivities in SLE patient groups. C) Forest plots of odds ratios with 95 % confidence intervals obtained with Fisher’s exact test for specific ANA reactivity with the indicated PB groups. Details are shown in Table 1. D) Level of U1-Sm/RNP-IgG in SLE patients split according to PB/M ratio. E) PB/M ratio in SLE patients split according to Sm/RNP-IgG reactivity. F) Heatmap with hierarchical clustering of individual patients (columns) based on immunological features (serological parameters and PB/M ratio; rows). The Z-score was calculated for each row. Dendrograms on the columns and rows were obtained using hierarchical clustering. PB/ M grouping and clinical characteristics for each patient (column) are shown in the bars below the heatmap. Each dot indicates an individual, and the bars represent the median (A,D,E). P values were obtained using Mann-Whitney test (A,B,D,E), or Fisher’s exact test (C).

Next, we wished to address the question whether specific ANA reactivities were increased in patients with a high PB/M ratio. In line with increased levels of total IgG, SLE patients with a high PB/M ratio had higher levels of total ANA-IgG (Fig. 6A), and had a broader recognition profile in ANA (Fig. 6B). It should be noted that both groups displayed heterogeneity in both of these measurements. Nonetheless, these differences were less pronounced or absent when grouping patients based on the frequency or absolute numbers of PB in circulation (Fig. S11A–D). These findings were independently confirmed in cohort 2A (Fig. S12A). Serum IgG reactivities to 8 nuclear antigens were measured by ELISA. Surprisingly, a strong increase in specific Sm/RNP reactivity was observed in patients with a high PB/M ratio (Table 1, Fig. 6C–E). This association was only observed for Sm/RNP reactivity and not for the other ANAs. Again, these differences were much smaller or less significant when analyzing the PB frequency, Bmem frequency, or the absolute count of PB (Fig. S11E–G, Table S5 and S6). Multiple logistic regression for anti-Sm/RNP positivity with the different PB parameters revealed a significant relationship with the PB/M ratio. No such relationship was noted for the other PB parameters (Fig. S11H). RNP70-positivity most frequently co-occurred with SLE-specific antibodies against Sm and dsDNA (Fig. S11I), and patients with RNP antibodies in the absence of SLE-specific antibodies did not have a diagnosis of mixed connective tissue disease.

Both Sm reactivity and increased PB signatures have been previously associated with African-American (AA) race [11,30,31]. Importantly, the association between the presence of a high PB/M ratio and Sm/RNP antibodies was statistically significant in non-AA SLE patients (OR: 10.31 (1.935–47.96); p = 0.0047), displayed a trend for significance in AA SLE patients (OR: 4.56 (1,02–17,93; p = 0.0623), and was replicated in an independent cohort (cohort 2A; Table S7, Fig. S12B–D). Therefore, a high PB/M ratio, that reflects B cell hyperactivity in SLE, is specifically associated with Sm/RNP IgG autoantibodies.

We summarized our findings in a heatmap in which Z-scores were calculated after log-transformation for immunological parameters (all ANAs, and IgG:IgM ratio and PB/M ratios) in SLE patients (Fig. 6F). Unsupervised hierarchical clustering analysis of the rows confirms the co-occurrence of ANA reactivities within antigen groups (Sm/RNP, chromatin, SS-A/B), which are known to frequently coincide within patients. Moreover, clustering of the rows confirmed the correlation between PB/M and IgG:IgM ratio as measures of B cell hyperactivity. Clustering of the patients in columns further shows separation of the two SLE subgroups with a high and low PB/M ratio on the left and right side of the heatmap, respectively. Patients with a high PB/M ratio often co-exhibit Sm/RNP reactivity and high IgG:IgM ratios. This group of patients also contains most patients with active disease (SLEDAI-2k & clinical activity; shown below the heatmap). Demographic and clinical characteristics of these patients are described in Tables S1–S4.

The PB/M ratio thus identifies a biologically relevant subgroup of SLE patients which is characterized by IFN-driven B cell hyperactivity and hypergammaglobulinemia, and displays frequent Sm/RNP reactivity and is more likely to have clinically active disease.

## 4. Discussion

In this study we identified a distinct phenotype in a subgroup of SLE patients which is characterized by a high PB frequency relative to CD27^+^ memory B cells. Patients with a high PB/M ratio also displayed other alterations within the B cell compartment. Whereas the lower frequency of CD27^+^ memory B cells was predominantly related to a decrease in unswitched memory B cells, CD27^+^ switched memory B cells and pre-PB were more activated in these patients, suggesting an increased pro-pensity of switched memory cells to differentiate to PB. We found increased proliferation and a strong IFN signature in PB from these patients. In line with increased PB differentiation, class-switched antibodies in serum were increased suggesting the PB/M ratio signifies a hyperactive B cell response. Together, these results reveal a strong relationship between type I IFN and B cell hyperactivity, highly enriched in patients with hypergammaglobulinemia and Sm/RNP antibodies. This reveals a novel subgroup of SLE patients in which IFN may directly drive B cell differentiation and increased (auto)antibody production.

We observed that patients with a high PB/M ratio exhibit serological features of B cell hyperactivity, with a strong enhancement of class-switched immunoglobulins in serum and a broader ANA profile. Previous studies have reported hypergammaglobulinemia in SLE, in particular increased IgG, as well as low IgM levels [32–34]. Our data confirmed these findings and revealed that low IgM and high IgG and IgA are particularly found in patients with a high PB/M ratio. Surprisingly, hypergammaglobulinemia and the IgG:IgM ratio only related to the PB/M ratio but not to the frequency of PB or PB count. One study reported a higher absolute PB count in patients with specific ANAs (dsDNA, Sm, SS-A and SS-B) in the absence of an effect on total IgG, IgA or IgM [14]. Furthermore, changes in the PB signature in whole blood associated with changes in anti-dsDNA levels, but an association with hypergammaglobulinemia was not reported [35]. Our results now link changes in the B cell subset composition with hypergammaglobulinemia in human SLE, providing evidence that B cell hyperactivity may directly lead to increased immunoglobulin production.

Patients with a high PB/M ratio displayed increased activation of switched B cells, in two distinct subsets; within the switched memory (CD27+IgD−) compartment, both the proportion of CD21^lo^ as well as their expression level of activation markers was increased. Within the DN (CD27-IgD-) B cell compartment, an increase in CD21^lo^ cells (DN2) was found as well, and these cells had increased expression of a more restricted set of activation markers. We found a strong correlation between the frequency of CD21^lo^ cells within switched memory B cells and DN cells. CD21^lo^ B cells have long been recognized as players in SLE and autoimmunity [21–23]. Whereas several studies have shown that both DN2 and switched memory B cells have an altered phenotype in SLE, most focus has been placed on DN2 cells [23,36]. Several studies reported increased frequency of DN2 cells in SLE, in particular in patients with active disease [23,37]. In our study, an increased frequency of DN2 cells was less pronounced, and many patients with a high PB/M ratio or high %PB did not display an increase in DN2 cells. This suggests that PB expansion and hypergammaglobulinemia can be present without an expansion of DN2 cells. Different characteristics, such as different race/ethnicities, and clinical activity (relatively low disease activity and low frequency of lupus nephritis), between our cohorts and previous cohorts on DN2 cells could explain the different findings on the frequency of DN2 cells.

In the context of infection and vaccination, resting memory B cells can give rise to both CD27+CD21^lo^ and CD27^−^ CD21^lo^ B cells upon recall, even within the same clone [38]. Both DN2 cells (CD27^−^ CD21^lo^) and activated switched memory cells (CD27+CD21^lo^) have been reported to have a high propensity to differentiate into PB/PC [23,39]. Since we found increased activation within both subsets, hyperactivity within both CD27^−^ as well as CD27^+^ B cells can be simultaneously present in SLE.

Patients with a high PB/M ratio had increased frequencies of IgG1+ PB, and these cells displayed SHM rates comparable to those in switched (IgG1) memory B cells from SLE patients that we and others have observed (Fig. 4H and I) [12,40]. Furthermore, CD45RB expression in PB was high, and similar to that of CD27^+^ memory B cells. This marker has been proposed to enable the tracking of activated B cells and PB derived from CD45RB + memory B cells [28]. Therefore, together these results point to activated switched memory B cells as a likely contributor to PB expansion in SLE. As such, the response has features of IFN-driven re-activation of memory B cells which have been previously generated through a GC response [41,42]. While IFN can enhance in vitro PB differentiation from memory B cells [43], IFN also plays an important role in GC B cell responses [44]. The inability to study GC B cells in SLE patients is a limitation of our study.

Several studies have reported increased PB frequencies or counts in SLE [8,11–14,40]. Such studies used either flow cytometry, scRNAseq, or whole blood transcriptome, and revealed that SLE is characterized by a high frequency of PB, in particular in active disease. A recent study showed that CD19^−^ PB/PC are also expanded in SLE, and are hallmarked by increased survival in SLE compared to vaccinated healthy individuals [40]. As we did not include staining for CD138 or other PC markers, we could not identify CD19^−^ PC. In the transcriptome of our CD19^+^ PB, we did not observe an increased maturation and/or survival signature (data not shown), possibly reflecting a difference between CD19^+^ and CD19 ^−^ PC. However, our findings on other properties of PB in SLE (polyclonal BCR, IgG skewing, IFN signature) is in line with previous studies [40, 45]. Our data, that were replicated in an independent cohort, suggest that the ratio of PB to CD27^+^ memory B cells is more characteristic of SLE, than the PB frequency alone, and that this phenotype relates to an IFN signature. Importantly, our results have shown that the PB/M ratio is associated with serological features of B cell hyperactivity.

Besides a correlation of the PB/M ratio with total immunoglobulins, we found a very strong association with Sm/RNP autoantibodies. Whether Sm/RNP antibodies arise as a consequence of enhanced PB differentiation from switched B cells or Sm/RNP positivity leads to a higher PB/M ratio remains to be determined. Sm/RNP antibodies can give rise to an IFN signature [46–48] which could subsequently cause increased PC differentiation from memory B cells [49]. However, other studies report an association of IFN with other ANA reactivities or the breadth of the ANA response regardless of specificity [50,51]. Therefore, it remains to be determined whether Sm/RNP antibodies give rise to B cell hyperactivity and a high PB/M ratio, or the other way around. It is also plausible that they are part of a feedforward loop in which they amplify each other. The frequent co-occurrence of anti-RNP antibodies with anti-Sm antibodies suggests that different components of the Sm/RNP complex are targeted in the PB/M high patient group, distinct from the isolated RNP-reactivity frequently observed in mixed connective tissue disease [52].

Higher IgG levels [53], a higher PB signature [11], Sm/RNP antibodies [30,31], and higher disease activity [54,55] have been associated with African-American ancestry in SLE patients, though these features can be present in SLE patients of any descent. In our study, the association between PB/M ratio and Sm/RNP reactivity was replicated in an independent European cohort, and was present in both AA and non-AA patients from the US cohort. Thus, though the hyperactive B cell phenotype, characterized by a high PB/M ratio, is more commonly observed in AA patients, it is also present in non-AA patients, and as such, represents a distinct biological patient phenotype regardless of race.

The relationship of a high PB/M ratio with disease activity suggests that B cell hyperactivity may play a role in disease pathogenesis and may thus guide the tailoring of B cell targeted therapies. Importantly, disease activity seemed to be higher across all clinical domains in patients with a high PB/M ratio. However, our cohorts were of limited sample size, and require larger studies to determine if the phenotype associates with specific disease manifestations. Thus far, our results suggest that immunological pathways associate with a range of clinical features, and that these may supplement clinical symptoms or manifestations in providing ways for patient stratification. The strong association with Sm/RNP reactivities indicates that serum autoantibody profile in combination with determining the B cell distribution (PB and CD27^+^ memory B cells) can be used to determine the degree of B cell hyperactivity and may be used for patient stratification in clinical trials, in particular those targeting global B cell activation, PB differentiation and type I IFN.

## Supplementary Material

1

## Figures and Tables

**Table 1:** Frequency of ANA reactivities in SLE patients with low versus high PB/M ratios from cohort 1. Positivity of ANA-IgG for each indicated antigen was determined using ELISA. Anti-chromatin refers to all patients positive for 1 or more of the antigens dsDNA, Nucleosomes, and histones. Anti-Sm/RNP refers to all patients positive for 1 or more of the antigens Sm, and RNP70. Anti-SS-A/B refers to all patients positive for 1 or more of the antigens SS-A, and SS-B (all patients with SS-B were also positive for SS-A).

	Low PB/M ratio (n = 30)	High PB/M ratio (n = 39)	Odds Ratio	p value
**Anti-chromatin**	17 (57 %)	21 (54 %)	0.89 (0.33–2.31)	>0.9999
dsDNA	15 (50 %)	21 (54 %)	1.17 (0.45–3.05)	0.8108
Nucleosomes	4 (13 %)	9 (23 %)	1.95 (0.60–6.21)	0.3650
Histones	5 (17 %)	9 (23 %)	1.50 (0.46–4.57)	0.5612
**Anti-Sm/RNP**	9 (30 %)	30 (77 %)	7.78 (2.59–20.83)	**0.0002** ***
Sm	6 (20 %)	20 (51 %)	4.21 (1.39–13.09)	0.0118 *
RNP70	2 (7 %)	20 (51 %)	14.74 (3.26–67.25)	**<0.0001** ****
U1-Sm/RNP	8 (27 %)	27 (69 %)	6.19 (2.14–16.08)	**0.0006** ***
**Anti-SS-A/B**	17 (57 %)	24 (62 %)	1.22 (0.45–3.29)	0.8056
SS-A	17 (57 %)	24 (62 %)	1.22 (0.45–3.29)	0.8056
SS-B	2 (7 %)	6 (15 %)	2.55 (0.56–13.02)	0.4509

P values were obtained using Fisher’s exact test.

## Appendix A. Supplementary data

Supplementary data to this article can be found online at https://doi.org/10.1016/j.jaut.2025.103438.
