# Supplementary material for: Interferon signatures fuel B cell hyperactivity and plasmablast expansion in systemic lupus erythematosus
Source: J Autoimmun. Author manuscript; Available in PMC 2026 May 26. (PMC13202177; doi:10.1016/j.jaut.2025.103438)
Supplement: 1 [file NIHMS2176183-supplement-1.pdf]

Supplementary material

- Supplementary Methods
- Supplementary Figures S1-S13
- Supplementary Tables S1-S9

SUPPLEMENTARY METHODS

Spectral flow cytometry data analysis

Raw spectral data was unmixed using Spectroflo software (Cytek), after which unmixed fcs files were analysed in OMIQ. A batch normalization control pooled PBMC sample was taken along in each experiment (4 experiments with fresh PBMCs, 1 experiment with frozen PBMCs). Live B cells were gated, after which expression of CD19, CD20, CD21, CD24, CD27, CD38, IgD, IgM, IgG and IgA across each experiment was normalized using CytoNorm (*1*). Subsequently, live B cells were clustered with FlowSOM (elbow metaclustering) using CD19, CD20, CD21, CD24, CD27, CD38, IgD, and IgM as input features. Expression of markers was analysed in UMAP and heatmaps to identify known B cell subsets (40). Fifteen FlowSOM metaclusters were obtained, from which 2 clusters were very similar in expresson patterns and were together designated as resting naive. One small cluster (<1%) was identified as non-B cells and excluded from downstream analysis, leaving 13 final B cell clusters. Using the same features as in FlowSOM, 500k subsamples cells across all samples were projected onto UMAP for visualization (neighbors = 100, mindist = 0.4). Cell counts and median fluorescence intensities (MFI) for each cluster and each sample were exported from OMIQ for further processing and statistical analysis. BST2 expression was normalized by subtracting the MFI of control cells (stained without BST2) and divided by the BST2 MFI of all B cells in the batch control PBMC sample.

Clustered heatmaps were made using scaled data obtained from OMIQ. Heatmaps for cluster identification was performed using median expression levels within subsets, Euclidean distance and Ward linkage in OMIQ. Hierarchical clustering displayed on heatmaps and PCA analysis for activation marker expression within subsets and patients were performed in R using pheatmap and prcomp respectively. Trajectory analysis was performed using Wanderlust in OMIQ using k=15 and 250 waypoints. After defining a small population of the most immature Tr1 cells, Wanderlust placed cells on a one-dimensional developmental trajectory, culminating in the most terminally differentiated PB cluster.

scRNAseq data analysis

Raw sequencing data was demultiplexed and aligned to the human genome (GRCh38-2020-A) using Cellranger software v4.0.0 (Experiment 1) and v5.0.1 (Experiment 2) (Chromium 10x). Analysis was performed with the Seurat v5.0.1 R package(*2*). Ig and TCR variable genes were removed to avoid bias in clustering/gene expression analysis by unique V gene usage. BCR repertoire was analysed separately (see below). Contaminating cells (non-PB) were removed following celltype prediction through the SingleR package using the Monaco immune reference dataset (13/5.5% removed) (*3, 4*). Low quality cells were removed based on a low number of detected genes and a high frequency of mitochondrial reads per cell (Experiment 1: >200 genes and <10% mt; Experiment 2: >2000 genes and <8% mt). Samples from run 2 were demultiplexed with the HTOdemux function in Seurat, followed by removal of

Negative/ambiguous and Doublet cells (4.3%) (*5*). In experiment 1, a small fraction of cells with the same barcode (<2%) was present in samples from different lanes (likely due to contamination), these were removed by retaining the cell with the highest read count. Final cell numbers for analysis were n=1290 cells from Experiment 1 and n=1197 cells from Experiment 2.

After pre-processing each Experiment individually, experiments were combined in Seurat, and integrated analysis was performed using anchor-based canonical correlation analysis (CCA) in the function IntegrateLayers. UMAP and FindNeighbors was performed using the first 20 integrated CCA dimensions. Clusters were identified by shared nearest neighbour (SNN) modularity optimization using FindNeighbors with CCA as reduction and 1:20 dimensions as well as FindClusters with resolutions ranging from 0.1 to 0.5 in 0.1 increments using FindClusters. By visual inspection and analysis of differentially expressed genes, and consistency across both experiments, clustering with a resolution of 0.1 (resulting in 2 clusters) was determined to best reflect distinct PB subsets. UMAP was generated using RunUMAP (n.neighbors = 100, min.dist = 0.4, spread = 1) and revealed good integration of both experiments.

Data were combined with JoinLayers for differential gene expression analysis. Data were normalized by the NormalizeData() function and scaled by the ScaleData() function. Covariate neighborhood analysis (CNA) was performed within Seurat using the rcna package (*6*) and visualized in UMAP space to analyse which neighborhoods were most associated with patient groups. Differential gene expression analysis was performed using FindMarkers (test.use = MAST, latent.vars = Experiment, logfc.threshold = 0.25). FindMarkers was performed twice, comparing the two identified PB clusters and comparing the two patient groups. One male patient in the Low PB/M group was excluded for comparison of the two patient groups as it led to identification of many male-specific transcripts in this group. This patient was included for subsequent analyses. Significant marker genes (adjusted p value<0.05) were exported in csv files and pathway analysis was performed using DAVID for gene ontology (GO) terms (biological pathways; BP). The resulting functional annotation chart was used in Cytoscape Enrichment Map for visualization of pathway enrichment (using cutoffs: FDR Q-value: 0.05; Overlap: 0.5; Test used: Overlap index) (*7*). Gene set enrichment analysis (GSEA) was performed with the ranked list of significant marker genes between groups using the GSEA function of the package ClusterProfiler. Source databases for GSEA were obtained from the GSEA molecular signature database (MsigDb) (*8*), and included hallmark cytokine gene sets, IFN-regulated transcription factors from dataset C. An additional dataset obtained from PBMCs stimulated with various IFNs and cytokines was used to generate a more detailed IFN signature (*9*)

5' RACE PCR

ANA+ B cells were stained as described (*10*), and ANA+ naïve (CD19+CD27-IgD+), double-negative (CD19+CD27-IgD-), and memory B cells (CD19+CD27+) were sorted from PBMCs of SLE patients used in scRNAseq experiment 2 using a FACS Aria (BD). 0.4-35k cells per population were lysed in RLT buffer (Qiagen) with 10 uL/mL 2-ME (Merck) after which RNA was isolated using RNeasy Microprep. cDNA was generated and amplified by incubating 8 uL RNA with Oligo-dT30VN (2.5 uM) and dNTPs (2.5 uM) for 3 min. at 72°C. All primers were ordered from IDT and sequences are provided in Table S10. Subsequently, first-strand buffer (Takara), Betaine BioUltra (1 M, Sigma-Aldrich), DTT (5 mM, Takara), recombinant RNase inhibitor (1 U/uL, Takara), SMARTScribe reverse transcriptase (5 U/uL, Takara), Template-

Switching Oligo (TSO, 1 uM) was added per sample. Samples were incubated for 90 min at 42°C, followed by 10 cycles of 2 min at 50°C and 2 min at 42°C, followed by incubation for 15 min at 72°C.
5’ RACE PCR products for Ig heacy chains (IgM, IgG, IgA) were generated using an adapted protocol for low cDNA amount based on the Anchoring Reverse Transcription of Immunoglobulin Sequences and Amplification by Nested PCR (*11, 12*). 2 µL of cDNA was added to a combination of Phusion Flash High-Fidelity PCR Master Mix, nuclease-free H_2O, SA forward primer (200 nM, SA.PCR_2) and either one of Ig-specific reverse primers (40 nM, IgM.PCR, IgG.PCR, IgA.PCR). Mixtures were incubated for 2 min at 98°C, followed by 40 cycles of 1 sec at 98°C, 15 sec at 69°C and 15 sec at 72°C, and final extension for 1 min at 72°C. PCR products were purified using Qiaquick PCR purification kit (Qiagen) according to manufacturer’s instructions. Subsequently, 4 uL of each sample was barcoded by adding a mix of Phusion Flash High-Fidelity PCR Master Mix, nuclease-free H_2O, one of the SA forward barcode family primers (200 nM) and one of the IgH-specific reverse barcode family primers (200 nM). Mixtures were incubated for 2 min at 98°C, followed by 10 cycles of 5 sec at 98°C, 15 sec at 65°C and 30 sec. at 72°C, and final extension for 5 min at 72°C. Samples were loaded on a 1% Agarose gel, followed by excision of the bands and purification using the Nucleospin Gel & PCR cleanup kit (Bioké). Different isotypes from the same sample were pooled. PCR products were quantified using the Qubit dsDNA Quantitation kit high sensitivity (Thermo Fisher), and samples were pooled in relative amount to their original cell number. Pooled PCR products were sequenced on a 8M SMRT cell (PacBio Sequel II) by the Leiden Genome Technology Center.

BCR repertoire analysis

BCR repertoire was analysed using the scRNAseq data and the 5’RACE data. For the first scRNAseq dataset, filtered contig sequences for V(D)J were generated from the Gene Expression data using Trust4 (*13*). For the second scRNAseq dataset, enriched V(D)J libraries were aligned using Cellranger software to generate filtered contig sequences. For the 5’ RACE PCR, raw sequencing reads were processed using pRESTO in Python. Sequences were trimmed using trimqual (Quality score threshold 50 and window size 5), after which sequences with a minimum length of 400 were selected. High quality sequences (quality score ≥90) were selected. Sample barcodes were assigned using MaskPrimers. Duplicate sequences were concatenated using CollapseSeq. C regions were assigned using MaskPrimers. Sequences with a minimum read count of 2 were retained using SplitSeq.
The BCR sequences from all three datasets were used as input for IMGT High V-quest (version 1.9.4; IMGT/V-QUEST reference directory release: 202405-2 F+ORF+ in-frame P - With all alleles; with search for insertions and deletions; other settings default).
Output from IMGT High V-quest was subsequently processed using the Immcantation pipeline. First, AIRR databases were created from the IMGT input and output files using the function MakeDb.py imgt in pRESTO (Python). Clonal analysis was done using SCOPER in R. Productive sequences were selected (>99%). Nucleotide Hamming distance was calculated and normalized by junction length using distToNearest. Hamming distance threshold was calculated using the density method in findThreshold. The resulting threshold of 0.145 was used to identify clones using hierarchicalClones. Clonal abundance and diversity was determined using Alakazam after downsampling to 100 cells per patient (3 patients excluded). Results were confirmed using several degrees of downsampling. Clonal diversity was calculated using alphaDiversity at q=0 to q=8. V gene usage was calculated using the Alakazam

package. Germline sequences were created using the createGermlines function in Dowser after which somatic hypermutation was calculated using the observedMutations function in the SHazaM package.

RNA isolation and qPCR

CD19+CD20loCD27hiCD38hi PBs were sorted from PBMCs of SLE patients using a FACS Aria (BD). 1-30k cells per population were lysed in RLT buffer (Qiagen) with 10 uL/mL 2-ME (Merck) after which RNA was isolated using RNeasy Microprep. cDNA was synthesized using iScript (Biorad). qPCR was performed after pre-amplification using TaqMan PreAmp Master Mix (Thermo) and the Taqman assays mentioned below. qPCR was performed with TaqMan Fast Advanced Master Mix (Thermo) and multiplexed VIC and FAM Taqman assays (all from Applied Biosystems/Thermo): POLR2A VIC-MGB (Hs00172187_m1), ACTB VIC-MGB (Hs01060665_g1), IFI6 FAM-MGB (Hs00242571_m1), BST2 FAM-MGB (Hs00171632_m1). qPCR was run on a CFX Opus machine (Biorad) using recommended cycling conditions. Relative expression in each sample was determined as the 2^(delta Ct) using the average of both housekeeping genes (POLR2A and ACTB).

REFERENCES

1. S. Van Gassen, B. Gaudilliere, M. S. Angst, Y. Saeys, N. Aghaeepour, CytoNorm: A Normalization Algorithm for Cytometry Data. *Cytometry A* **97**, 268-278 (2020).
2. Y. Hao, S. Hao, E. Andersen-Nissen, W. M. Mauck, 3rd, S. Zheng, A. Butler *et al.*, Integrated analysis of multimodal single-cell data. *Cell* **184**, 3573-3587 e3529 (2021).
3. D. Aran, A. P. Looney, L. Liu, E. Wu, V. Fong, A. Hsu *et al.*, Reference-based analysis of lung single-cell sequencing reveals a transitional profibrotic macrophage. *Nat Immunol* **20**, 163-172 (2019).
4. G. Monaco, B. Lee, W. Xu, S. Mustafah, Y. Y. Hwang, C. Carre *et al.*, RNA-Seq Signatures Normalized by mRNA Abundance Allow Absolute Deconvolution of Human Immune Cell Types. *Cell Rep* **26**, 1627-1640 e1627 (2019).
5. M. Stoeckius, S. Zheng, B. Houck-Loomis, S. Hao, B. Z. Yeung, W. M. Mauck, 3rd *et al.*, Cell Hashing with barcoded antibodies enables multiplexing and doublet detection for single cell genomics. *Genome Biol* **19**, 224 (2018).
6. Y. A. Reshef, L. Rumker, J. B. Kang, A. Nathan, I. Korsunsky, S. Asgari *et al.*, Co-varying neighborhood analysis identifies cell populations associated with phenotypes of interest from single-cell transcriptomics. *Nat Biotechnol* **40**, 355-363 (2022).
7. D. Merico, R. Isserlin, O. Stueker, A. Emili, G. D. Bader, Enrichment map: a network-based method for gene-set enrichment visualization and interpretation. *PLoS One* **5**, e13984 (2010).
8. A. Subramanian, P. Tamayo, V. K. Mootha, S. Mukherjee, B. L. Ebert, M. A. Gillette *et al.*, Gene set enrichment analysis: a knowledge-based approach for interpreting genome-wide expression profiles. *Proc Natl Acad Sci U S A* **102**, 15545-15550 (2005).
9. M. D. Catalina, P. Bachali, N. S. Geraci, A. C. Grammer, P. E. Lipsky, Gene expression analysis delineates the potential roles of multiple interferons in systemic lupus erythematosus. *Commun Biol* **2**, 140 (2019).
10. J. Suurmond, Y. Atisha-Fregoso, E. Marasco, A. N. Barlev, N. Ahmed, S. A. Calderon *et al.*, Loss of an IgG plasma cell checkpoint in patients with lupus. *J Allergy Clin Immunol* **143**, 1586-1597 (2019).

11. M. T. Koning, S. M. Kielbasa, V. Boersma, H. P. J. Buermans, S. A. J. van der Zeeuw, C. A. M. van Bergen *et al.*, ARTISAN PCR: rapid identification of full-length immunoglobulin rearrangements without primer binding bias. *Br J Haematol* **178**, 983-986 (2017).
12. L. M. Slot, R. D. Vergroesen, P. F. Kerkman, E. Staudinger, S. Reijm, H. J. van Dooren *et al.*, Light chain skewing in autoantibodies and B-cell receptors of the citrullinated antigen-binding B-cell response in rheumatoid arthritis. *PLoS One* **16**, e0247847 (2021).
13. L. Song, D. Cohen, Z. Ouyang, Y. Cao, X. Hu, X. S. Liu, TRUST4: immune repertoire reconstruction from bulk and single-cell RNA-seq data. *Nat Methods* **18**, 627-630 (2021).

SUPPLEMENTARY FIGURES

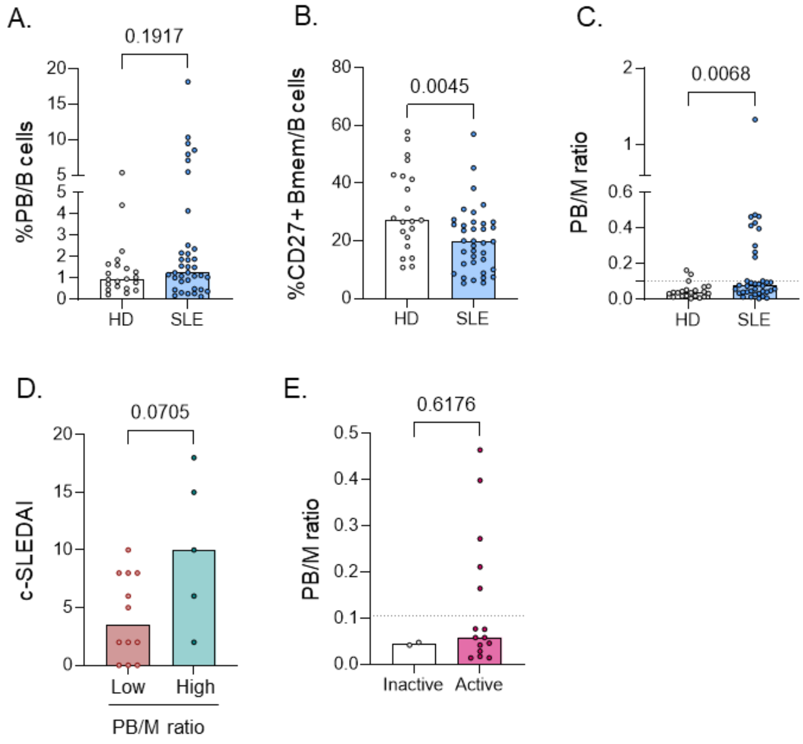

Figure S1: A high PB to memory B cell ratio characterizes a subgroup in SLE in cohort 2A.
B cell phenotypes of SLE patients (n=37) and healthy donors (n=21) were analysed using flow cytometry. Clinical data was available for 17 of these SLE patients. A-C) %PB, %CD27+ Bmem among total B cells, and PB/M ratio. D) c-SLEDAI in SLE patients with a low versus high PB/M ratio. E) PB/M ratio in patients with clinically inactive versus active (c-SLEDAI>0) disease.
Each dot indicates an individual, and the bars represent the median. P values were calculated using Mann-Whitney test.

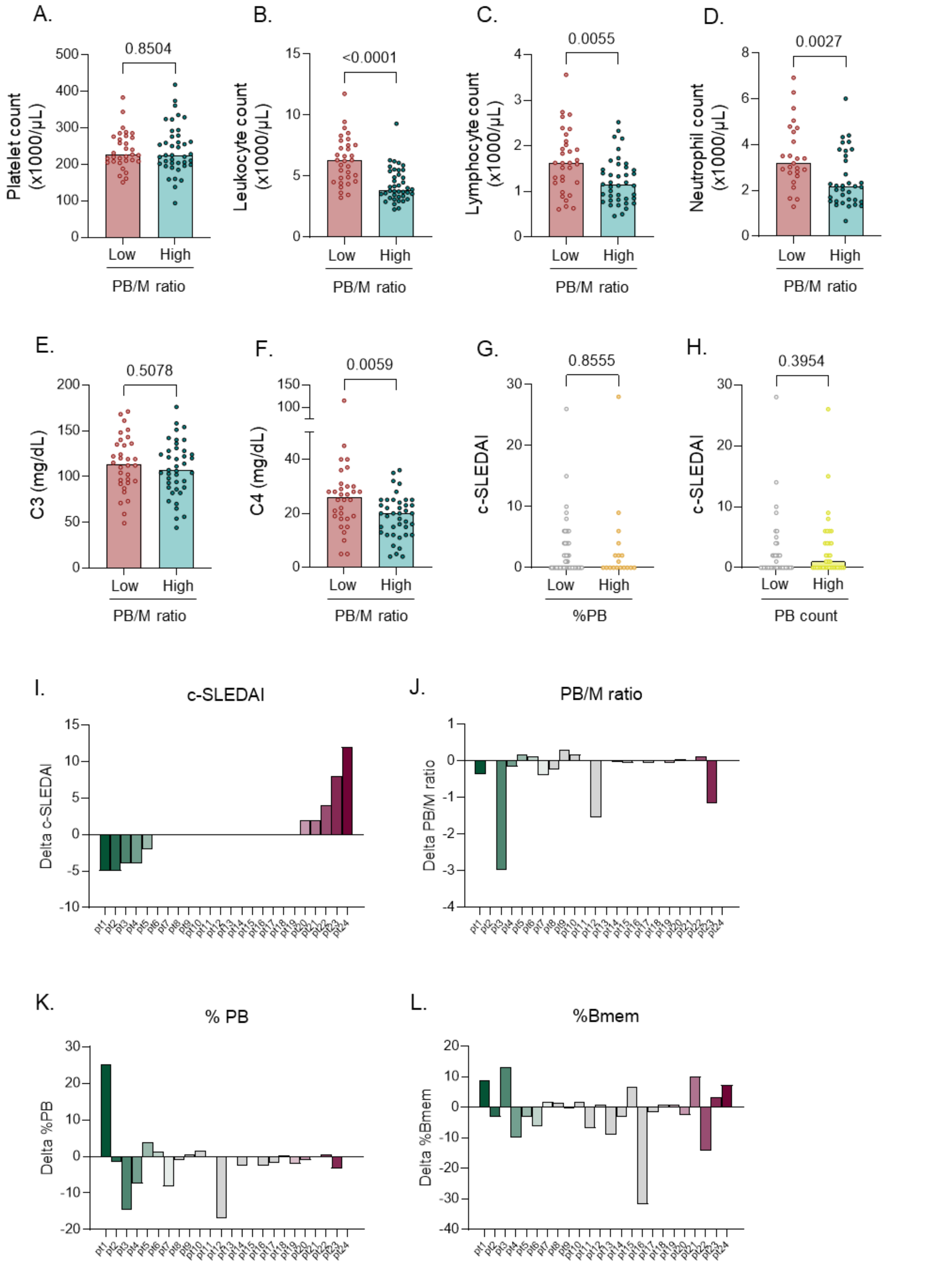

Figure S2: Additional analysis of disease activity in SLE patient groups in Cohort 1.
B cell phenotypes of SLE patients (n=72) and healthy donors (n=14) were analysed using flow cytometry. A-F) Differential blood cell counts and complement levels in SLE patients with a low versus high PB/M ratio. G,H) c-SLEDAI in SLE patients with a low versus high %PB or PB count. I-L) c-SLEDAI change over time and lack of association with changes in B cell subset percentages in SLE patients whose B cell phenotype was measured

twice (n=24). Patients were sorted based on delta c-SLEDAI (I), after which the delta PB/M ratio (J), delta %PB (K), and delta %CD27+ Bmem were plotted in the same order.
Each dot indicates an individual, and the bars represent the median. P values were calculated using Mann-Whitney test (A-H).

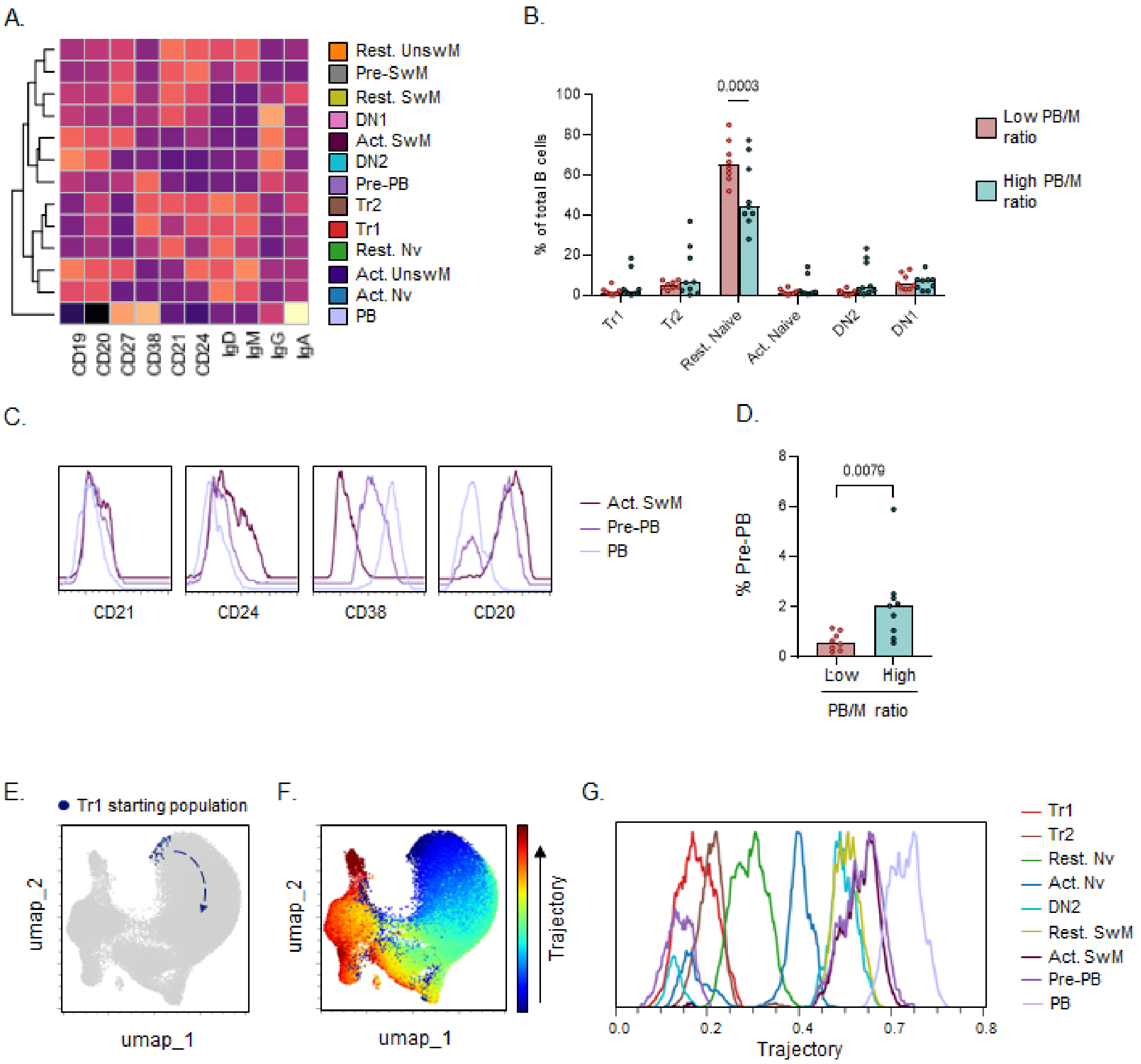

Figure S3: Additional spectral flow cytometry phenotyping in fresh PBMCs in Cohort 2C.
High dimensional spectral flow cytometry was used for detailed B cell phenotyping within fresh PBMCs from SLE patients (SLE; n=17). A) Heatmap showing expression levels of markers (columns) for each FlowSOM cluster (rows). Z-score was calculated using the median expression among all samples. B) % of CD27- B cell populations SLE patients with a low versus high PB/M ratio. C) Histograms of key markers defining the Pre-PB cluster. D) % Pre-PB SLE patients with a low versus high PB/M ratio. E) Trajectory analysis using Wanderlust. Cells indicated as Tr1 in left UMAP were the starting population. F) Resulting trajectory projected on UMAP. G) Location of main B cell subsets on the Wanderlust trajectory, displayed as a histogram of maximized cell counts per cluster.
Each dot indicates an individual, and the bars represent the median. P values were calculated using Mann-Whitney test (D), or Two-way ANOVA with FDR posthoc test (B).

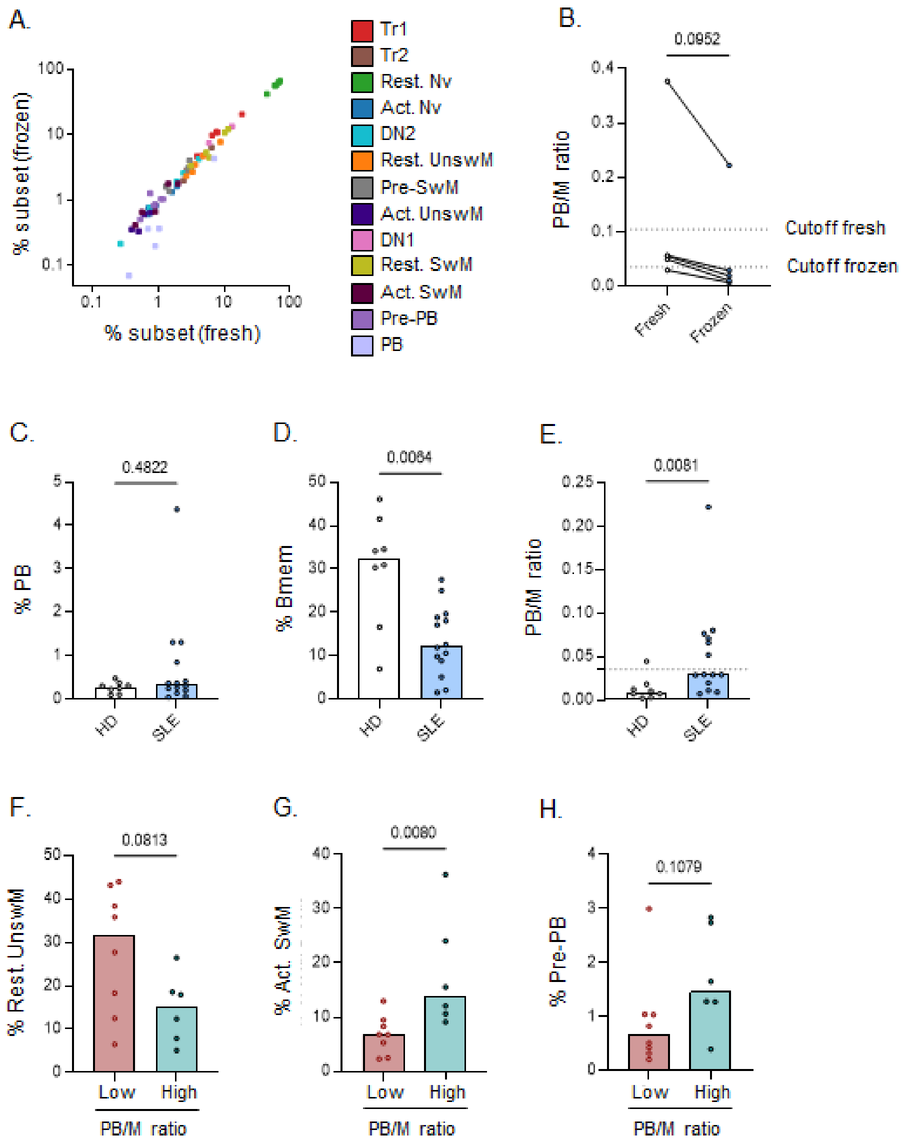

Figure S4: Replication of spectral flow cytometry phenotyping in frozen PBMCs in Cohort 2D.

High dimensional spectral flow cytometry was used for detailed B cell phenotyping within frozen PBMCs from SLE patients (n=15) and healthy donors (HD; n=10). For 5 SLE patients, paired analysis of fresh PBMCs was also performed. A) Correlation of the percentage of each cluster in paired frozen and fresh samples, showing only the PB cluster is affected by freezing. B) Paired analysis of the PB/M ratio in fresh and frozen samples. The cutoff for each dataset is indicated and was based on the distribution in healthy donors (Q3+1.5*IQR). C-E) %PB, %CD27+ Bmem, and PB/M ratio in healthy donors compared to SLE patients. F-H) %Rest UnswM, Act SwM, Pre-PB SLE patients with a low versus high PB/M ratio.

Each dot indicates an individual, and the bars represent the median. P values were calculated using Mann-Whitney test (C-H).

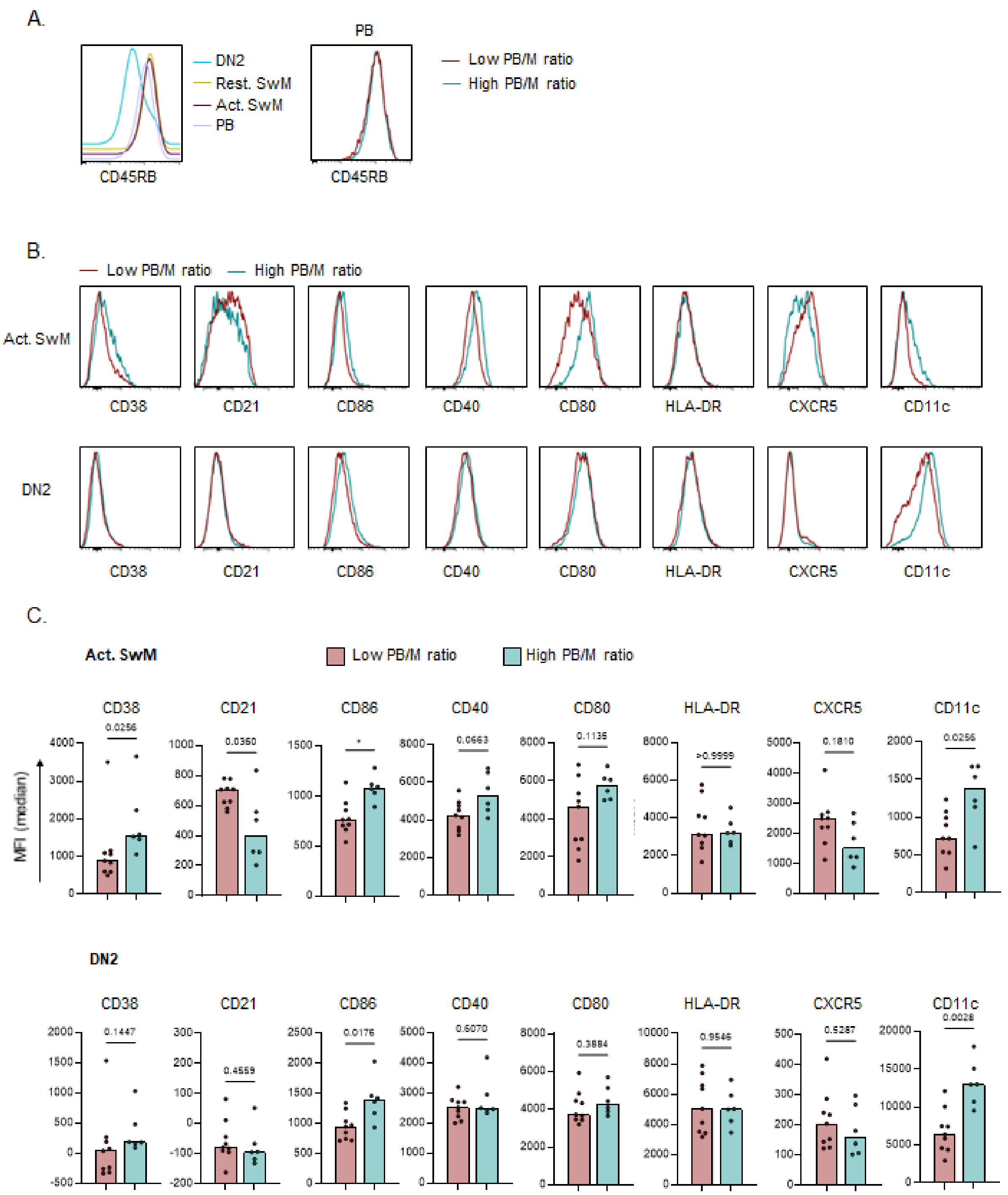

Figure S5: Additional spectral flow cytometry phenotyping in frozen PBMCs in Cohort 2D.

High dimensional spectral flow cytometry was used for detailed B cell phenotyping within frozen PBMCs from SLE patients (n=15) and healthy donors (HD; n=10). A) CD45RB expression in several switched B cell subsets (left) and comparison of CD45RB expression in PB from SLE patient groups. B) Histograms showing expression of activation markers within Act SwM (top row) and DN2 cells (bottom row) in SLE patients with a low versus high PB/M ratio. C) Expression level of activation markers between the two patient groups in Act SwM (top row) and DN2 cells (bottom row).

Each dot indicates an individual, and the bars represent the median. P values were calculated using Mann-Whitney test (C).

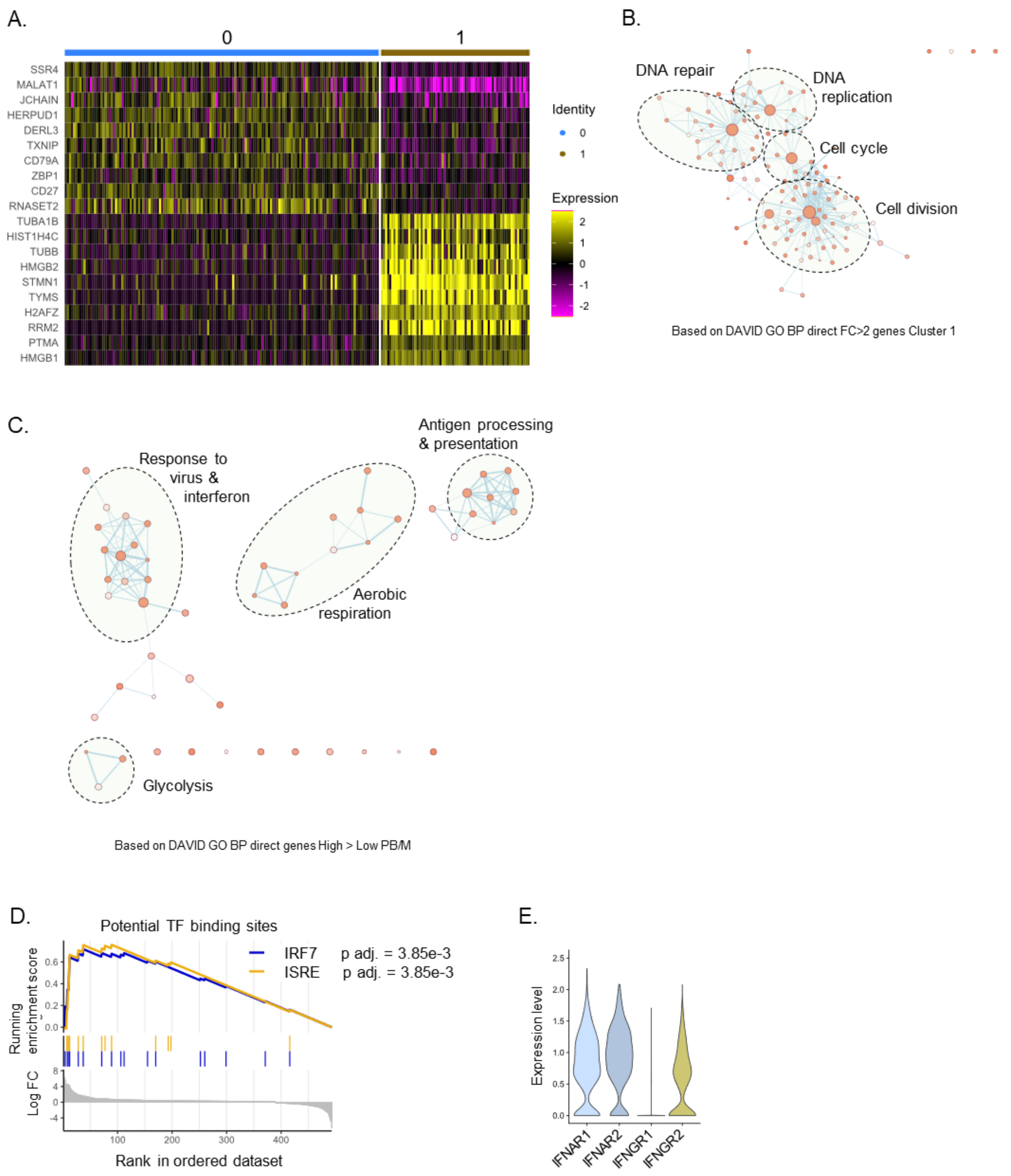

Figure S6: Increased proliferation and IFN signature underly PB expansion in patients with a high PB/M ratio. scRNA-seq was performed on sorted PB from 9 SLE patients, in 2 independent experiments (cohort 1 and cohort 2B). A) Heatmap of top 10 differentially expressed genes (adjusted p value <1*10e-10, highest fold change) between patients with a low versus high PB/M ratio. 150 cells from cluster 0 and 75 cells from cluster 1 are shown. B) Clustering of enriched pathways in cluster 1 determined using DAVID functional enrichment analysis and visualized using enrichmentMAP. C) Clustering of enriched pathways in patients with a high PB/M ratio as in B. D) GSEA for regulatory target gene sets and predicted transcription factor binding sites of IFN-regulated factors using differentially expressed genes (adjusted p value <0.05; ordered by log fold change) between the two patient groups. E) Expression level of IFN receptor subunits.

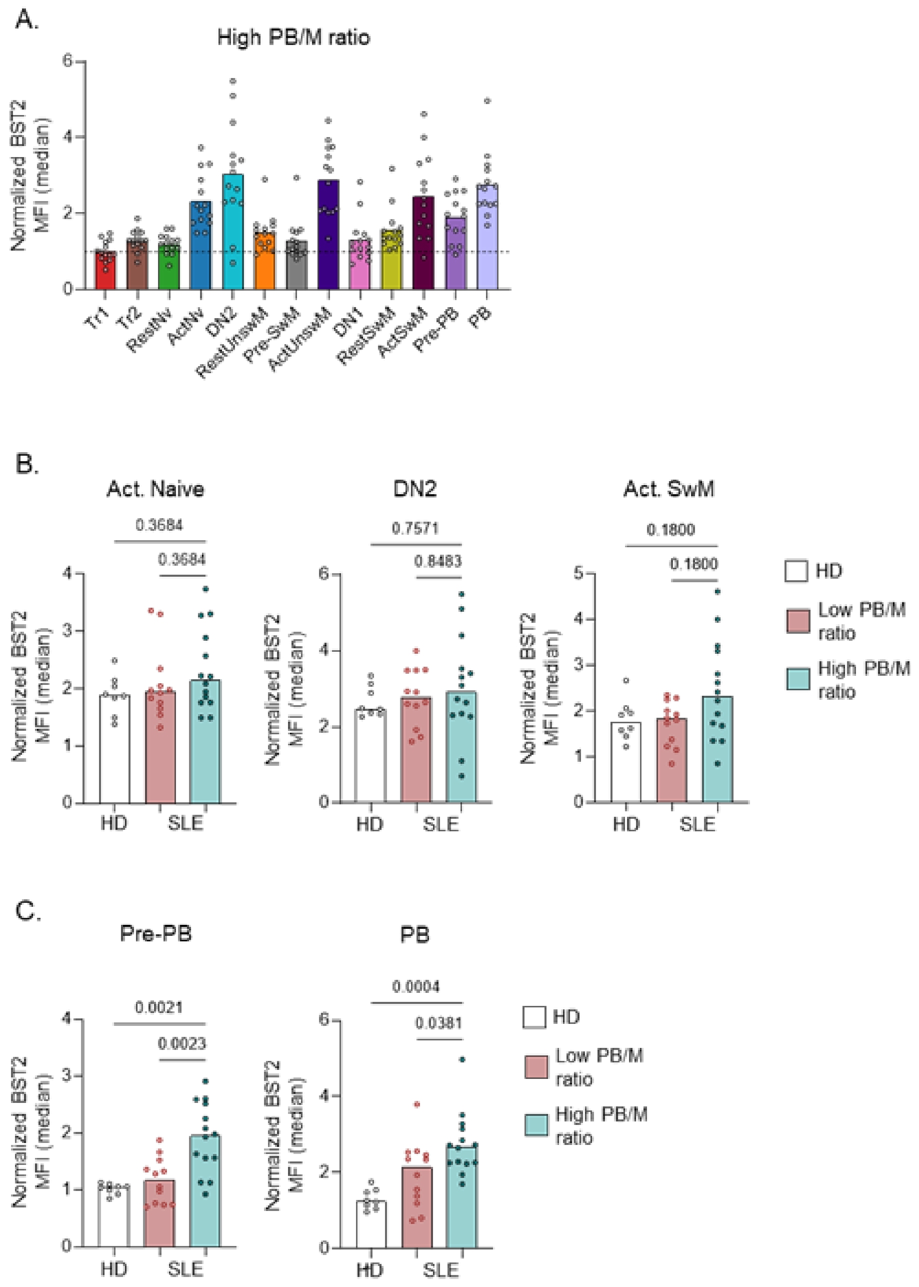

Figure S7: BST2 protein expression in B cell subsets in Cohort 2D.

High dimensional spectral flow cytometry was used for analysis of BST2 expression within B cell subsets from SLE patients (n=26) and healthy donors (HD; n=8). Additional data on subset definitions is provided in Figure 2, S3 and S4. A) Normalized expression of BST2 in B cell subsets from patients with a high PB/M ratio. B,C) Comparison of normalized BST2 expression between patient groups and HD in subsets with high BST2 expression.

Each dot indicates an individual, and the bars represent the median. P values were calculated using Kruskal-Wallis with FDR posthoc test (B,C).

A.

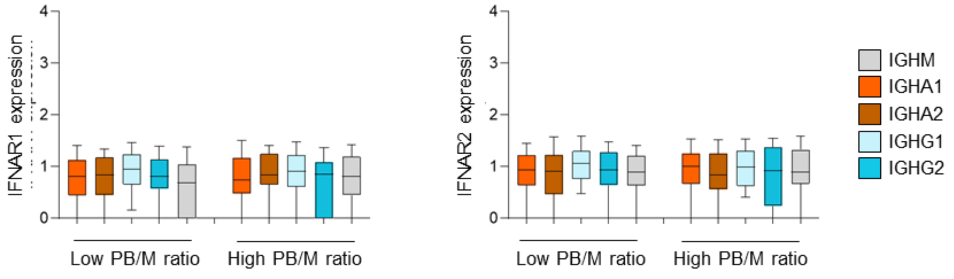

B.

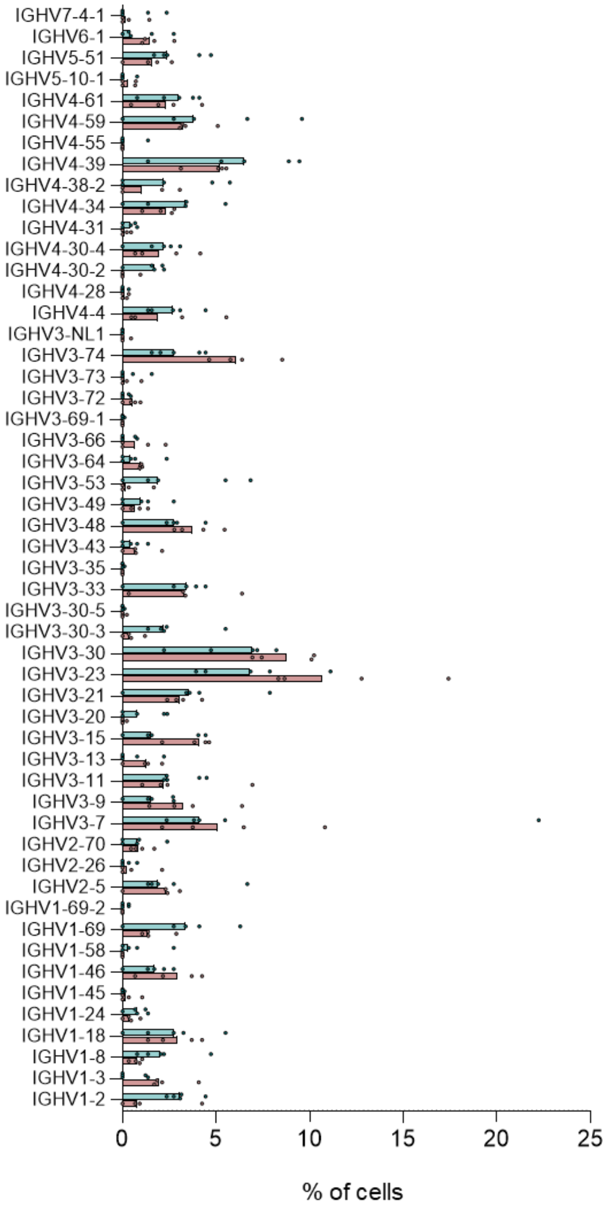

Figure S8: IFNAR expression by isotype and V gene distribution

BCR repertoire was determined using results from scRNA-seq (Figure 3) from 9 SLE patients, in 2 independent experiments (cohort 1 and cohort 2B). A) scRNAseq expression level of IFNAR1 and IFNAR2 in cells from each

IGH isotype/subclass, separated by patient group. IGHD, IGHG3, and IGHG4 cells were not analysed due to low number of cells (Figure 4A). B) Expression of heavy chain V genes, expressed as percentage of total PB.

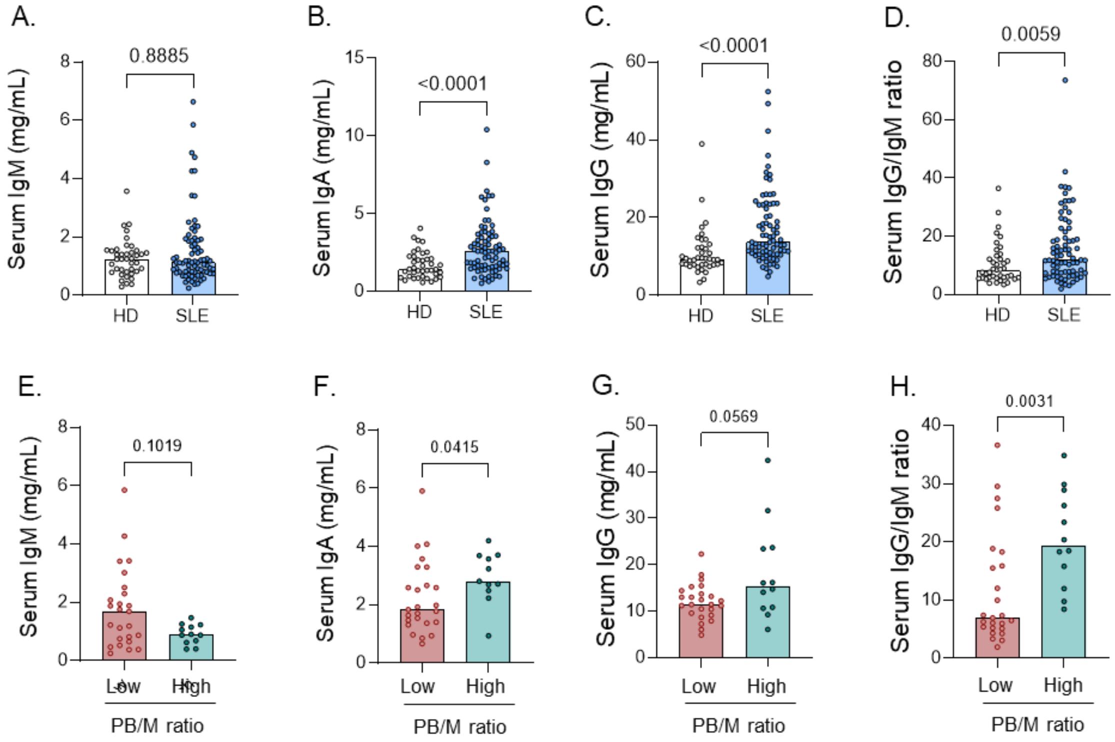

Figure S9: B cell hyperactivity results in hypergammaglobulinemia (replication cohort).
Serum antibodies were measured in SLE patients (cohort 2; n=77) and healthy donors (n=40) using ELISA. A-H) Serum levels of total IgG, IgA, and IgM, and IgG:IgM ratio in SLE patients and healthy donors (A-D). SLE patients for which flow cytometry data was available were split into two groups based on PB/M phenotype shown in Figure 1 (cohort 2A; n= 37; E-H).
Each dot indicates an individual, and the bars represent the median (A-H,J). P values were obtained using Mann-Whitney test (A-H).

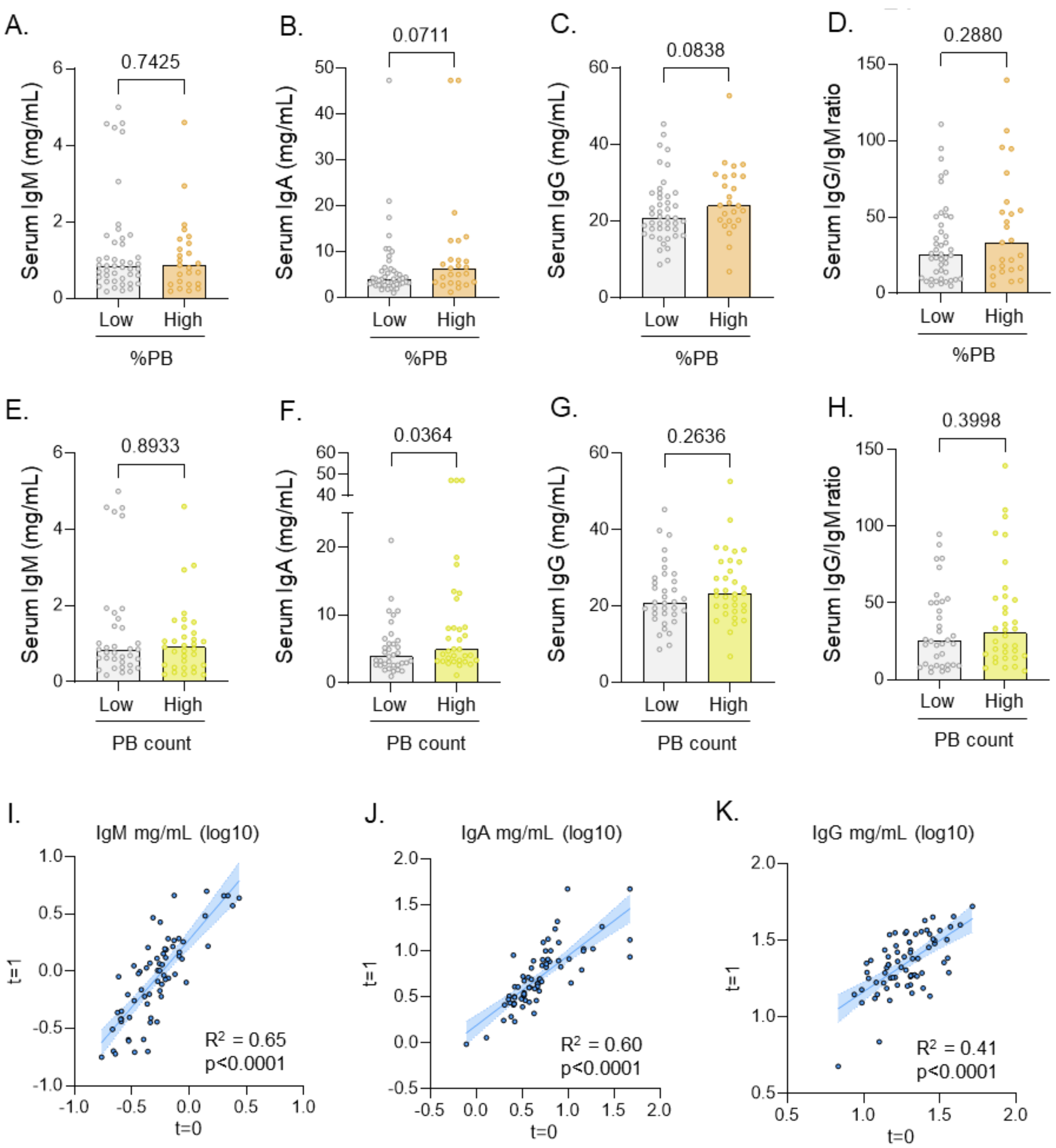

Figure S10: Hypergammaglobulinemia correlates less with PB count and %PB in Cohort 1.
Serum antibodies were measured in SLE patients (n=69) and healthy donors (n=40) using ELISA. SLE patients were split into two groups based on %PB and PB count. A-H) Serum levels of total IgG, IgA, and IgM, and IgG:IgM ratio in SLE patients split according to %PB (A-D) or PB count (E-H). I-K) Correlation of serum immunoglobulin levels in SLE patients measured at two timepoints ~1 year apart (n=72).
Each dot indicates an individual, and the bars represent the median (A-H). P values were obtained using Mann-Whitney test (A-H), or simple linear regression (I-K).

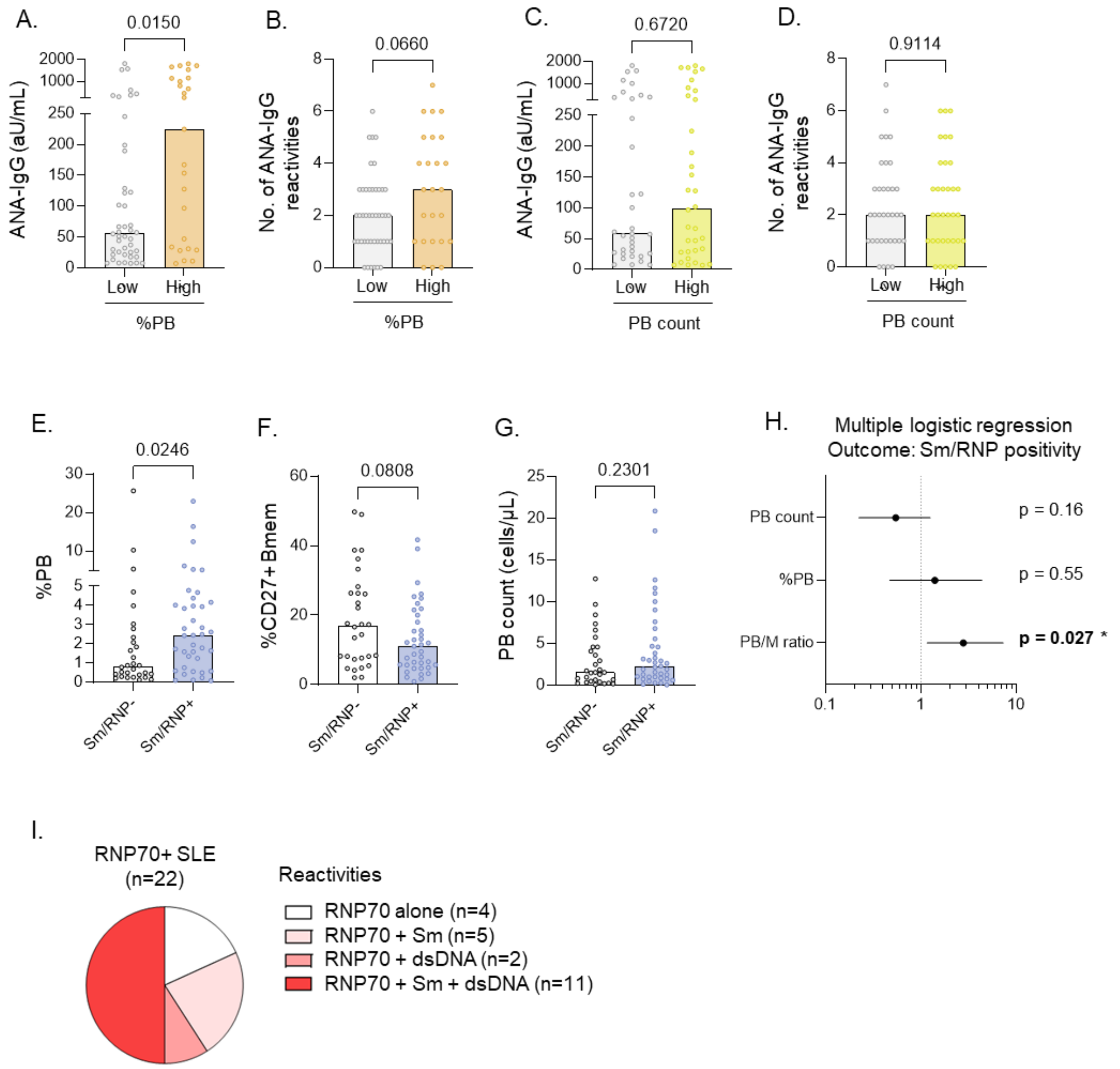

Figure S11: Sm/RNP reactivity correlates less with PB count and %PB and additional analysis of RNP reactivity in Cohort 1

Serum autoantibodies were measured in SLE patients (n=69) using ELISA. Healthy donors (n=40) were used as negative controls. Autoantibody reactivities were categorized into three main antigen groups: chromatin (dsDNA, nucleosomes, histones); Sm/RNP (Sm, RNP70, U1-RNP complex), SS-A/B (SS-A, SS-B). SLE patients were split into two groups based on PB phenotype shown in Figure 1. A-D) Level of ANA-IgG and number of reactivities in SLE patients split according to %PB (A,B) and PB count (C,D). E-G) %PB, %Bmem, and PB count in SLE patients split according to Sm/RNP-IgG reactivity. H) Forest plot of multiple logistic regression model with Sm/RNP reactivity as outcome, and the Z-score of PB count, %PB, PB/M ratio, and cohort as predicting variables. I) Number of SLE-specific autoantibody co-occurrence in anti-RNP70+ SLE patients (n=22).

Each dot indicates an individual, and the bars represent the median (A-G). P values were obtained using Mann-Whitney test (A-G), or multiple logistic regression (H).

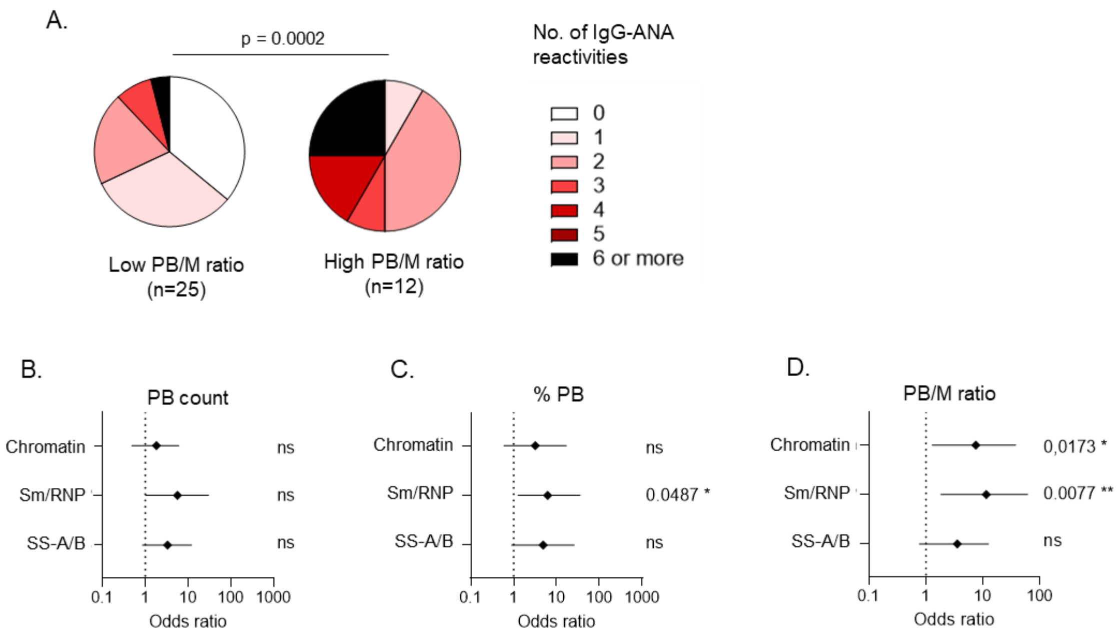

Figure S12: B cell hyperactivity is specifically associated with the presence of Sm/RNP autoantibodies in Cohort 2A.

Serum autoantibodies were measured in SLE patients (n=37) using ELISA. Healthy donors (n=40) were used as negative controls. Autoantibody reactivities were categorized into three main antigen groups: chromatin (dsDNA, nucleosomes, histones); Sm/RNP (Sm, RNP70, U1-RNP complex), SS-A/B (SS-A, SS-B). SLE patients were split into two groups based on PB phenotype shown in Figure 1. A) Number of IgG-ANA reactivities in SLE patient groups. B-D) Forest plots of odds ratios with 95% confidence intervals obtained with Fisher's exact test for specific ANA reactivity with the indicated PB groups. Details are shown in Table S7.

P values were obtained using Mann-Whitney test (A), or Fisher's exact test (B-D).

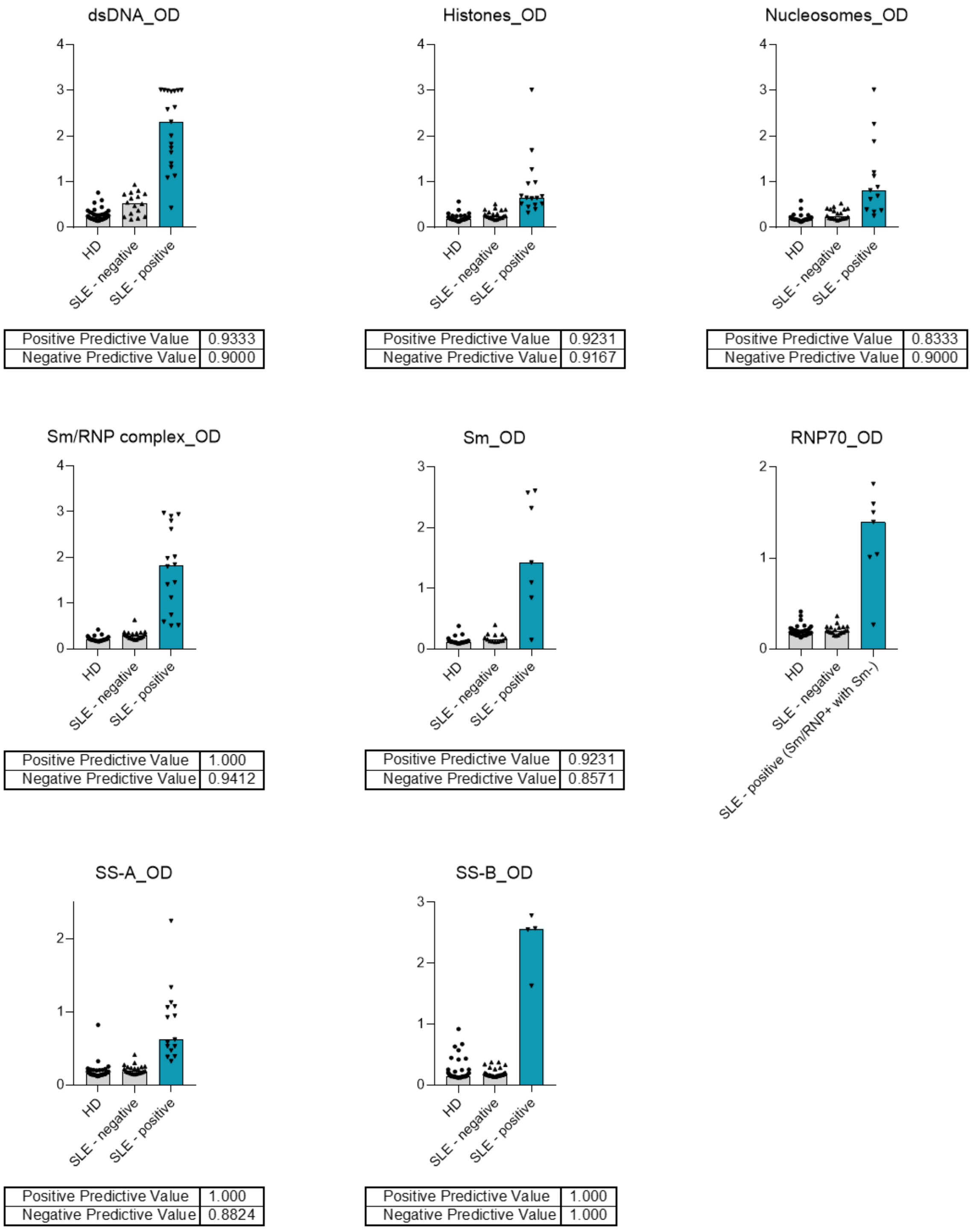

dsDNA_OD

Positive Predictive Value	0.9333
Negative Predictive Value	0.9000

Histones_OD

Positive Predictive Value	0.9231
Negative Predictive Value	0.9167

Nucleosomes_OD

Positive Predictive Value	0.8333
Negative Predictive Value	0.9000

Sm/RNP complex_OD

Positive Predictive Value	1.000
Negative Predictive Value	0.9412

Sm_OD

Positive Predictive Value	0.9231
Negative Predictive Value	0.8571

SS-A_OD

Positive Predictive Value	1.000
Negative Predictive Value	0.8824

SS-B_OD

Positive Predictive Value	1.000
Negative Predictive Value	1.000

Figure S13: Validation of in-house ANA ELISAs. SLE patients negative or positive for the respective antigen were selected based on commercial ELISA or clinical diagnostic assays (Sm). Forty healthy donors were used to calculate a cutoff (mean + 4x SD) after which positive and negative predictive values were calculated using Fisher's exact test. For RNP70, positive predictive value could not be calculated as the exact status of RNP70 reactivity was unknown (samples included Sm-RNP+ samples that were Sm-negative).

	Low PB/M ratio (n=32)	High PB/M ratio (n=40)	p value
Age (yrs)	41 (21-64)	41 (19-70)	0.6745
Male/female (% female)	1/31 (97%)	7/33 (83%)	0.0684
Ethnicity (self-identified)			
African-American	12 (38%)	25 (63%)	0.0570
Hispanic	13 (41%)	9 (23%)	0.1251
Caucasian	3 (9.4%)	5 (13%)	0.7254
Asian	4 (13%)	0 (0%)	0.0350
Other	0 (0%)	1 (2.5%)	>0.9999
Disease duration (yrs)	10 (1-21)	10 (0-36)	0.4822
Age at diagnosis (yrs)	30 (9-56)	25 (7-66)	0.1666
Current medication			
PDN	15 (47%)	21 (53%)	0.8128
HCQ	28 (88%)	31 (78%)	0.3611
Immunosuppressants[1]	14 (44%)	19 (48%)	0.8145

Table S1: Patient characteristics in PB/M low and PB/M high groups in Cohort 1 (Feinstein)

The characteristics shown here are obtained during their initial assessment. All parameters are shown as the median and range or number and frequency within the patient group. None of the patients had used cytoxan or rituximab in the preceding 12 months.

Statistical testing was done using Mann Whitney U test for linear parameters, chi-square test for categorical parameters with more than 2 categories (ethnicity) and Fisher’s exact test for categorical parameters with 2 categories (male/female and medication).

[1] Immunosuppressants include Mycophenolate mofetil, Azathioprine and Methotrexate.

PDN: Prednisone, HCQ: Hydroxychloroquine.

	Low PB/M ratio (n=25)	High PB/M ratio (n=12)	p value
Age (yrs)	50 (22-76)	47 (25-76)	0.8792
Male/female (% female)	1/24 (96%)	1/11 (92%)	>0.9999
Disease duration (yrs)	18 (2-39)	21 (4-41)	0.3550
Age at diagnosis (yrs)	31 (11-65)	30 (13-40)	0.6713
Current medication			
PDN	3 (12%)	6 (50%)	0.0355
HCQ	19 (76%)	10 (83%)	>0.9999
Immunosuppressants[1]	6 (24%)	6 (50%)	0.1138

Table S2: Patient characteristics in low PB/M and high PB/M groups in Cohort 2B (Leiden)
The characteristics shown here are obtained during their initial assessment. All parameters are shown as the median and range or number and frequency within the patient group. None of the patients had used cytoxan or rituximab in the preceding 12 months.
Statistical testing was done using Mann Whitney U test for linear parameters, and Fisher's exact test for categorical parameters with 2 categories (male/female and medication).
[1] Immunosuppressants include Mycophenolate mofetil, Azathioprine and Methotrexate.
PDN: Prednisone, HCQ: Hydroxychloroquine.

	Low PB/M ratio (n=32)	High PB/M ratio (n=40)	p value
Inactive (SLEDAI-2k = 0)	12 (38%)	5 (13%)	**0.0293 ***
Low (SLEDAI-2k = 1-4)	14 (44%)	19 (48%)	
Moderate (SLEDAI-2k = 5-8)	5 (16%)	8 (20%)	
High (SLEDAI-2k >8)	1 (3%)	8 (20%)	

Table S3: Disease activity in SLE patients from low PB/M and high PB/M groups in Cohort 1.
P value was obtained using Chi-square test.

	Low PB/M ratio (n=32)	High PB/M ratio (n=40)	p value
Renal	2 (6%)	7 (18%)	0.2821

Mucocutaneous	7 (22%)	14 (35%)	0.2988
Musculoskeletal	3 (9%)	7 (18%)	0.4955
Hematological	0 (0%)	6 (15%)	**0.0304 ***
Other	0 (0%)	4 (10%)	0.1216

Table S4: Clinical symptoms in SLE patients from low PB/M and high PB/M groups in Cohort 1. P value was obtained using Fishers exact test.

	%PB low (n=44)	%PB high (n=25)	Odds Ratio	p value
**Anti-chromatin**	25 (57%)	13 (52%)	0.8233 (0.2939-2.322)	0.8027
dsDNA	23 (52%)	13 (52%)	0.9891 (0.3569-2.783)	>0.9999
Nucleosomes	5 (11%)	8 (32%)	3.671 (1.119-11.77)	0.0538
Histones	6 (14%)	8 (32%)	2.980 (0.9692-9.832)	0.1171
**Anti-Sm/RNP**	21 (48%)	18 (72%)	2.816 (0.9689-7.431)	0.0766
Sm	13 (30%)	13 (52%)	2.583 (0.8907-6.867)	0.0760
RNP70	8 (18%)	14 (56%)	5.727 (1.947-15.83)	**0.0026 ****
U1-Sm/RNP	17 (39%)	18 (72%)	4.084 (1.390-10.95)	**0.0119 ***
**Anti-SS-A/B**	26 (59%)	15 (60%)	1.038 (0.3704-2.697)	>0.9999
SS-A	26 (59%)	15 (60%)	1.038 (0.3704-2.697)	>0.9999
SS-B	4 (9.1%)	4 (16%)	1.905 (0.5078-7.014)	0.4480

Table S5: Frequency of ANA reactivities in SLE patients with a low and high %PB in Cohort 1. Positivity of ANA-IgG for each indicated antigen was determined using ELISA. Anti-chromatin refers to all patients positive for 1 or more of the antigens dsDNA, Nucleosomes, and histones. Anti-Sm/RNP refers to all patients positive for 1 or more of the antigens Sm, and RNP70. Anti-SS-A/B refers to all patients positive for 1 or more of the antigens SS-A, and SS-B (all patients with SS-B were also positive for SS-A).
P values were obtained using Fisher’s exact test.

	PB count low (n=35)	PB count high (n=34)	Odds Ratio	p value
**Anti-chromatin**	22 (63%)	16 (47%)	0.5253 (0.1915-1.345)	0.2301
dsDNA	21 (60%)	15 (44%)	0.5263 (0.1943-1.337)	0.2316
Nucleosomes	5 (14%)	8 (23%)	1.778 (0.5649-5.531)	0.5401
Histones	7 (20%)	7 (21%)	1.037 (0.3340-3.220)	>0.9999
**Anti-Sm/RNP**	17 (49%)	22 (65%)	1.941 (0.7524-4.925)	0.2270
Sm	11 (31%)	15 (44%)	1.722 (0.6402-4.295)	0.3261
RNP70	7 (20%)	15 (44%)	3.158 (1.043-8.526)	**0.0406 ***
U1-Sm/RNP	15 (43%)	20 (59%)	1.905 (0.7510-5.157)	0.2316
**Anti-SS-A/B**	23 (66%)	18 (53%)	0.5870 (0.2271-1.537)	0.3319
SS-A	23 (66%)	18 (53%)	0.5870 (0.2271-1.537)	0.3319
SS-B	5 (14%)	3 (8.8%)	0.5806 (0.1448-2.546)	0.7096

Table S6: Frequency of ANA reactivities in SLE patients with a low and high PB count in Cohort 1. Positivity of ANA-IgG for each indicated antigen was determined using ELISA. Anti-chromatin refers to all patients positive for 1 or more of the antigens dsDNA, Nucleosomes, and histones. Anti-Sm/RNP refers to all patients positive for 1 or more of the antigens Sm, and RNP70. Anti-SS-A/B refers to all patients positive for 1 or more of the antigens SS-A, and SS-B (all patients with SS-B were also positive for SS-A).
P values were obtained using Fisher's exact test.

	Low PB/M ratio (n=25)	High PB/M ratio (n=12)	Odds Ratio	p value
**Anti-chromatin**	10 (40%)	10 (83%)	7.50 (1.29-37.78)	**0.0173 ***
dsDNA	8 (32%)	10 (83%)	10.63 (1.77-53.42)	**0.0051 ****
Nucleosomes	3 (12%)	3 (25%)	2.44 (0.48-11.76)	0.3666
Histones	4 (16%)	5 (42%)	3.75 (0.73-14.61)	0.1161
**Anti-Sm/RNP**	2 (8%)	6 (50%)	11.50 (1.84-61.50)	**0.0077 ****
Sm	2 (8%)	6 (50%)	11.50 (1.84-61.50)	**0.0077 ****
RNP70	0 (0%)	2 (17%)	Inf (1.01-inf)	0.0991
U1-Sm/RNP	1 (4%)	6 (50%)	24.00 (2.47-283.0)	**0.0023 ****
**Anti-SS-A/B**	9 (36%)	8 (67%)	3.56 (0.77-12.57)	0.1575
SS-A	9 (36%)	8 (67%)	3.56 (0.77-12.57)	0.1575
SS-B	4 (16%)	6 (50%)	5.25 (1.16-20.51)	**0.0486 ***

Table S7: Frequency of ANA reactivities in SLE patients from low PB/M and high PB/M groups in the replication cohort 2B. Positivity of ANA-IgG for each indicated antigen was determined using ELISA. Anti-chromatin refers to all patients positive for 1 or more of the antigens dsDNA, Nucleosomes, and histones. Anti-Sm/RNP refers to all patients positive for 1 or more of the antigens Sm, and RNP70. Anti-SS-A/B refers to all patients positive for 1 or more of the antigens SS-A, and SS-B (all patients with SS-B were also positive for SS-A).
P values were obtained using Fisher's exact test.

Table S8: Conventional flow cytometry & FACS antibodies

Antigen	Fluorochrome	Clone	Company	Catalog Number
CD3	BV510	UCHT1	Biolegend	300447
CD3	eFluor506	UCHT1	eBioscience	69-0038-42
CD14	BV510	M5E2	Biolegend	301841
CD14	eFluor506	61D3	eBioscience	69-0149-42
CD19	BB700	SJ25C1	BD Biosciences	566396
CD19	PE-Cy7	HIB19	Biolegend	302215
CD19	PerCp-eF710	SJ25C1	eBioscience	46-0198-42
CD20	AF700	2H7	Biolegend	302322
CD20	eFluor450	2H7	eBioscience	48-0209-42
CD20	FITC	2H7	Biolegend	302304
CD27	BV421	M-T271	BD Biosciences	562513

Antigen	Fluorochrome	Clone	Company	Catalog Number
CD27	PE	0323	eBiosciences	12-0279-42
CD38	BV711	HIT2	Biolegend	303528
CD38	PE	HB7	BD	345806
CD38	PE-eFluor610	HIT2	eBioscience	61-0389-42
CD56	eF506	TULY56	eBioscience	48-0566-42
IgA	PE-Vio770	IS11-8E10	Miltenyi	130-114-003
IgD	AF700	IA6-2	Biolegend	348229
IgD	PE-CF594	IA6-2	BD Biosciences	562540
IgG	FITC	G18-145	BD Biosciences	555786
IgG	BV605	G18-145	BD Biosciences	563246
IgM	FITC	MHM-88	Biolegend	314506
IgM	PerCpCy5.5	MHM-88	Biolegend	314511

Table S9: Spectral flow cytometry antibodies

Antigen	Fluorochrome	Clone	Company	Catalog Number
CD1c (BDCA1)	PE	AD5-8E7	Miltenyi	120-000-889
CD1d	PE-Cy7	51.1	Biolegend	350309
CD3	eFluor 506	UCHT1	eBioscience	69-0038-42
CD11c	FITC	3.9	Biolegend	301604
CD14	eFluor 506	61D3	eBioscience	69-0149-42
CD19	BV570	HIB19	Biolegend	302236
CD20	BUV395	2H7	BD	563782
CD21	BUV805	B-ly4	BD	742008
CD24	BUV496	ML5	BD	741143
CD27	APC-Fire810	QA17A18	Biolegend	393214
CD32B/C	AF647	4F5	Biolegend	Custom labeled by Biolegend
CD38	BUV563	HB7	BD	741446
CD40	PerCpCy5.5	5C3	Biolegend	334315
CD45RB	BUV615	MT4 (6B6)	BD	751482
CD49D	BV711	9F10	Biolegend	304331

Antigen	Fluorochrome	Clone	Company	Catalog Number
CD69	PE-Fire640	FN50	Biolegend	310959
CD70	PE	Ki-24	BD Biosciences	555835
CD80	APC-R700	L307.4	BD	565157
CD86	BV650	IT2.2	Biolegend	305427
CD138	BV711	MI15	Biolegend	356521
CD183 (CXCR3)	AF488	G025H7	Biolegend	353709
CD183 (CXCR3)	BV785	G025H7	Biolegend	353737
CD197 (CCR7)	APC-Fire750	G043H7	Biolegend	353245
CD317 (BST2)	PE-Cy7	RS38E	Biolegend	348415
CXCR5	BV750	J252D4	Biolegend	356941
HLA-DR	PE-Fire810	L243	Biolegend	307683
IgD	BV480	IA6-2	BD	566138
IgM	SuperBright436	SA-DA4	eBioscience	62-9998-42
IgG	BV421	G18-145	BD	562581
IgA	VioBlue	IS11-8E10	Miltenyi	130-113-479
Ki67	PerCp-eF70	20Raj1	eBioscience	46-5699-42
S1PR4	AF488	1012512	R&D systems	FAB10321G

Table S10: Primer sequences for 5' RACE PCR

Primer name	Sequence (5' → 3')	Barcode
Oligo-dT30VN	AAGCAGTGGTATCAACGCAGAGTACT30VN	
TSO	AAGCAGTGGTATCAACGCAGAGTACATrGrG+G	
SA.PCR_2	CTTAAGCAGTGGTATCAACGCAGAGTACATG	
IgM.PCR	CACAGGAGACGAGGGGGAAAAGGG	
IgG.PCR	GGAAGGTGTGCACGCCGCTGGTC	
IgA.PCR	CGCTCCAGGTCACACTGAGTGG	
**Barcoding**		
F-bc01_SA.pcr_2	[F-bc]-TTAAGCAGTGGTATCAACGCAGAGTACA	GGTAGATGT
F-bc02_SA.pcr_2	..	GGTAGCAAG
F-bc03_SA.pcr_2	..	GGTAGGACC
F-bc04_SA.pcr_2	..	GGTAGTAGA
F-bc05_SA.pcr_2	..	GGTAGAGTT
F-bc06_SA.pcr_2	..	GGTAGCCTC
F-bc07_SA.pcr_2	..	GGTAGGCAT
F-bc08_SA.pcr_2	..	GGTAGTCAA
F-bc09_SA.pcr_2	..	GGTAGACGC
F-bc10_SA.pcr_2	..	GGTAGCGAT
F-bc11_SA.pcr_2	..	GGTAGGTAA
F-bc12_SA.pcr_2	..	GGTAGTGCG
R-bc01_m.bc_2	[R-bc]-GTTGGGGCGGATGCACTCC	CCATCATGT
R-bc02_m.bc_2	..	CCATCGAAT
R-bc03_m.bc_2	..	CCATCGATA
R-bc04_m.bc_2	..	CCATCGTGT
R-bc01_g.bc_2	[R-bc]-AGTAGTCCTTGACCAGGCAGCC	CCATCATGT
R-bc02_g.bc_2	..	CCATCGAAT
R-bc03_g.bc_2	..	CCATCGATA
R-bc04_g.bc_2	..	CCATCGTGT
R-bc01_a.bc_2	[R-bc]-GCGGGAAGACCTTGGGG	CCATCATGT
R-bc02_a.bc_2	..	CCATCGAAT
R-bc03_a.bc_2	..	CCATCGATA
R-bc04_a.bc_2	..	CCATCGTGT

Table S11: Cohorts used for each main figure and its replication

**Finding**	Main figure	Main cohort	Replication figure	Replication cohort
High PB to memory B cell ratio in SLE	Figure 1	Cohort 1	Figure S1	Cohort 2B
Activation in switched B cells	Figure 2	Cohort 2D	Figure S4 & S5	Cohort 2E
Increased proliferation and IFN signature in PB	Figure 3A-J	Cohort 1 & 2C	Two cohorts were combined for an integrated analysis; main findings were observed in both cohorts/each patient (e.g. Figure 3J)	
BST2 expression in PB	Figure 3K-M	Cohort 2D	Figure S7	Cohort 2E
IgG skewing and polyclonal expansion	Figure 4	Cohort 1 & 2C	Two cohorts were combined for an integrated analysis; IgG skewing was validated by flow cytometry in cohort 2D (Figure 4C,D)	
Hypergammaglobulinemia	Figure 5	Cohort 1	Figure S9	Cohort 2A
Sm/RNP reactivity	Figure 6	Cohort 1	Figure S12	Cohort 2B
